# Liver sinusoidal endothelial cells rely on oxidative phosphorylation but avoid processing long-chain fatty acids in their mitochondria

**DOI:** 10.1186/s11658-024-00584-8

**Published:** 2024-05-09

**Authors:** Patrycja Kaczara, Izabela Czyzynska-Cichon, Edyta Kus, Anna Kurpinska, Mariola Olkowicz, Kamila Wojnar-Lason, Marta Z. Pacia, Olena Lytvynenko, Myriam Baes, Stefan Chlopicki

**Affiliations:** 1 Jagiellonian University, Jagiellonian Centre for Experimental Therapeutics (JCET), Bobrzynskiego 14, 30-348 Krakow, Poland; 2 Jagiellonian University Medical College, Department of Pharmacology, Grzegorzecka 16, 31-531 Krakow, Poland; 3 KU Leuven, Department of Pharmaceutical and Pharmacological Sciences, Laboratory of Cell Metabolism, 3000 Leuven, Belgium

**Keywords:** LSEC, Mitochondrial respiration, Glycolysis, Peroxisomes, l-carnitine, Palmitic acid

## Abstract

**Background:**

It is generally accepted that endothelial cells (ECs), primarily rely on glycolysis for ATP production, despite having functional mitochondria. However, it is also known that ECs are heterogeneous, and their phenotypic features depend on the vascular bed. Emerging evidence suggests that liver sinusoidal ECs (LSECs), located in the metabolically rich environment of the liver, show high metabolic plasticity. However, the substrate preference for energy metabolism in LSECs remains unclear.

**Methods:**

Investigations were conducted in primary murine LSECs in vitro using the Seahorse XF technique for functional bioenergetic assays, untargeted mass spectrometry-based proteomics to analyse the LSEC proteome involved in energy metabolism pathways, liquid chromatography-tandem mass spectrometry-based analysis of acyl-carnitine species and Raman spectroscopy imaging to track intracellular palmitic acid.

**Results:**

This study comprehensively characterized the energy metabolism of LSECs, which were found to depend on oxidative phosphorylation, efficiently fuelled by glucose-derived pyruvate, short- and medium-chain fatty acids and glutamine. Furthermore, despite its high availability, palmitic acid was not directly oxidized in LSEC mitochondria, as evidenced by the acylcarnitine profile and etomoxir’s lack of effect on oxygen consumption. However, together with l-carnitine, palmitic acid supported mitochondrial respiration, which is compatible with the chain-shortening role of peroxisomal β-oxidation of long-chain fatty acids before further degradation and energy generation in mitochondria.

**Conclusions:**

LSECs show a unique bioenergetic profile of highly metabolically plastic ECs adapted to the liver environment. The functional reliance of LSECs on oxidative phosphorylation, which is not a typical feature of ECs, remains to be determined.

**Supplementary Information:**

The online version contains supplementary material available at 10.1186/s11658-024-00584-8.

## Introduction

Endothelial cells (ECs) distributed throughout the vascular system share many features, including producing specific mediators that regulate vascular homeostasis and prevent thrombosis, inflammation or organ injury [1–3]. However, the endothelium is a highly heterogeneous organ, and some aspects of the EC phenotype strongly depend on the vascular bed, representing a specific adaptation to the microenvironment of a given organ [4–6]. Liver sinusoidal ECs (LSECs) have particularly unique morphological and functional features, including fenestrae that regulate the passage of macromolecules, especially lipoproteins [7], and a high clearance capacity to remove various blood-borne macromolecules, such as oxidised lipoproteins, immune complexes or some viruses [8]. Importantly, proper LSEC function maintains liver homeostasis, while LSEC dysfunction contributes to various liver pathologies. Indeed, LSECs regulate not only liver perfusion but also liver regeneration after acute injury, liver fibrosis, liver inflammation, systemic infections [9] and metabolic processes [10]. Therefore, LSECs are emerging as a potential therapeutic target in liver diseases. However, little is currently known about the energy metabolism of LSECs.

It seems generally accepted that, regardless of the vascular bed, ECs rely mainly on glycolysis for ATP production [11, 12] despite having functional mitochondria. Glycolysis is required for EC proliferation and migration during angiogenesis [12] and contributes to the endothelial and vascular inflammatory response [13]. ECs contain relatively few mitochondria compared with other cells [14]. However, mitochondria play important roles in signalling, calcium homeostasis, haem turnover and apoptosis regulation [15, 16]. Furthermore, the oxidation of fatty acids (FAs) in mitochondria is essential for biomass production during vascular sprouting [17] and for redox homeostasis in quiescent ECs [18].

Given the liver microenvironment (rich in metabolic substrates) and the unique functional features of LSECs, the question arises about whether LSECs have adapted to this rich environment through preferences towards specific metabolic substrates or rely on glycolysis such as other types of ECs. More than 30 years ago, Spolarics et al. demonstrated in freshly isolated LSECs in a suspension that their primary energy sources were glutamine and FAs but not glycolysis [19]. Interestingly, Dudek et al. recently demonstrated that mitochondrial respiration with various metabolic fuels also prevails over glycolysis in LSECs attached to the surface after cell isolation and suggested that oxidative phosphorylation is important for scavenging and antigen-cross-presenting immune functions of LSECs [20]. However, they did not analyse individual energy sources but focused on the mechanisms of LSEC-dependent priming of cluster of differentiation 8 (CD8)^+^ T cells.

Surprisingly, we previously found that prolonged exposure in vivo to a high-fat diet (HFD) only transiently impaired mitochondrial function in isolated LSECs [21]. This effect was observed in the early phase of metabolic dysfunction-associated steatotic liver disease (MASLD), together with a mild pro-inflammatory response, but without affecting the LSEC fenestrae, suggesting that LSECs might be resistant to HFD-induced endothelial pathology. These findings indicate that the substrate preference of LSECs may be distinct from other types of ECs, likely due to their prominent adaptive capacity to the metabolic substrate-rich microenvironment of the liver [21]. These results were also surprising in the context of the known effects of excessive FAs on other types of ECs, which develop impaired nitric oxide- and prostacyclin-dependent function and mitochondrial dysfunction [22–25]. Even a short-term HFD treatment induces severe endothelial dysfunction in the aorta [26] and impairs coronary endothelial function [27] that persists during extended HFD administration, leading to cardiovascular pathology.

To explain this apparent paradox of divergent responses of aortic and liver ECs to excessive FAs, we explored the bioenergetic profile of primary murine LSECs based on functional responses and proteomic analysis in this study. Specifically, we studied substrate plasticity and the role of glucose-derived pyruvate and short- and medium-chain FAs (SCFAs and MCFAs) as preferable substrates for oxidative phosphorylation and ATP production in LSECs. Our results demonstrate that LSECs represent an unusual type of highly metabolically plastic ECs adapted to the liver environment.

## Materials and methods

Details of key resources are provided in Additional file 2.

### Animal model

Male C57BL/6J mice aged 6–12 weeks were obtained from the Medical University of Bialystok (Poland) and housed in colony cages in a temperature-controlled environment (22 ± 2 °C) with a 12/12 h light/dark cycle. Mice had free access to food and water. All animal procedures were conducted according to the Guide for the Care and Use of Laboratory Animals of the European Union (Directive 2010/63/EU).

### LSECs isolation

Mouse LSECs were isolated from multiple animals as previously described [21]. Briefly, the blood was flushed from the livers through the portal vein with a perfusion buffer (37 °C), followed by the digestion of connective tissue with a collagenase solution (Liberase TM). The obtained cell suspension was differentiated by a series of low-speed centrifugations and gradient density sedimentation in 25–50% Percoll. Finally, the LSEC-specific CD146-based immunoseparation on magnetic MACS MicroBeads was used to purify the LSEC fraction. In Additional file 1: Fig. S1 presented are LSEC marker proteins identified using a proteomic analysis, selected based on a review work by Nagy et al. [6]. Primary LSECs were incubated overnight in Endothelial Cell Growth Medium-2 (EGM-2; supplemented with 2% fetal bovine serum [FBS]) at 37 °C and 5% CO_2_ and used for experiments within 24 h of isolation.

### Cell lines and cell culture

The human aortic endothelial cell line (HAECs, cat. no. CC-2535) was purchased from Lonza and cultured in EGM-2. The human lung microvascular endothelial cell line (hLMVECs, cat. no. 540-05a) was purchased from Cell Application and cultured in Microvascular Endothelial Cell Growth Medium (MECGM). The human breast cancer adenocarcinoma MCF-7 cell line (cat. no HTB-22) and the breast cancer MDA-MB-231 cell line (cat. no HTB-26) were purchased from the American Type Culture Collection. MCF-7 cells were cultured in Eagle medium supplemented with 2 mM l-glutamine, 1% minimal essential medium non-essential amino acid solution, 0.8 mg/L of human insulin solution and 10% FBS). MDA-MB-231 cells were cultured in RPMI 1640, supplemented with 2 mM l-glutamine and 10% FBS (Additional file 2).

### Analysis of energy metabolism

Primary LSECs were seeded at 70,000 per well into Seahorse XFe96 plates in EGM-2 medium and incubated overnight at 37 °C and 5% CO_2_. Experiments using the Seahorse analyser (Seahorse Bioscience, N. Billerica, MA, USA) were carried out in two types of assay media based on (1) bicarbonate-free Agilent Seahorse XF Base Medium Minimal Dulbecco’s modified Eagle’s medium (DMEM) or (2) Krebs-HEPES buffer (KHB; 111 mM NaCl, 4.7 mM KCl, 1.25 mM CaCl_2_, 2 mM MgSO_4_ · 7H_2_O and 1.2 mM NaH_2_PO_4_ and 5 mM HEPES; pH 7.4) and supplemented accordingly to specific experiments (the details are described in the figures legends) freshly on the day of the experiment. Both oxygen consumption rate (OCR) and extracellular acidification rate (ECAR) were analysed in the assay media based on unbuffered DMEM; only the OCR was analysed in the assay buffers based on KHB. On the day of the experiment, the cells were washed twice with a suitable assay medium and placed for 1 h of incubation at 37 °C without CO_2_ before starting the measurements. For the experiments with bovine serum albumin (BSA)-conjugated FAs, BSA was used as a control. Standard mitochondrial modulators concentrations were optimised in preliminary experiments and used as follows: oligomycin (an ATP synthase inhibitor) at 1 µg/mL without BSA and 2 µg/mL with BSA, carbonyl cyanide 4-[trifluoromethoxy]phenylhydrazone (FCCP, a mitochondrial uncoupler) at 2 µM without BSA and 5 µM with BSA and rotenone with antimycin A (R/A; inhibitors of complex I and complex III, respectively) at 0.5 µM without BSA and 1 µM with BSA (Additional file 2).

### ATP measurement

Primary LSECs were seeded at 70,000 per well into a 96-well plate in EGM-2 and incubated overnight at 37 °C and 5% CO_2_. The cells were treated with oligomycin (1 µg/mL) and/or iodoacetic acid (IAA; a glyceraldehyde-3-phosphate dehydrogenase [GAPDH] inhibitor; 20 µM) or dimethyl sulfoxide (DMSO) as the vehicle control for 60 min followed by the ATPlite 1step Luminescence Assay System (Perkin Elmer, Waltham, MA) according to the manufacturer’s protocol.

### Fluorescence microscopy

Primary LSECs were plated into 96-well Falcon imaging microplates (Corning, USA) at 100,000 per well. The mitochondria of living LSECs were labelled with the MitoTracker Green FM probe. Briefly, cells were gently washed with warm (37 °C) Dulbecco’s phosphate-buffered saline (DPBS) containing calcium and magnesium and incubated with 100 nM MitoTracker Green FM solution in EGM-2 for 30 min (37 °C). Hoechst 33342 (1:2000) was used for nuclei staining. Then, the cells were washed with DPBS containing calcium and magnesium (37 °C) and immediately imaged with an Axio Imager.A2 fluorescent microscope (Carl Zeiss, Germany).

### Liquid chromatography–tandem mass spectrometry analysis of acyl-carnitine species

Primary LSECs were seeded at 2 million per well in six-well plates in EGM-2 medium and incubated overnight at 37 °C and 5% CO_2_. On the day of the experiment, the cells were washed twice with fatty acid oxidation (FAO) buffer (KHB containing 2.5 mM glucose and 50 µM l-carnitine). Cells were untreated or pre-incubated for 15 min with etomoxir (ETO; a carnitine palmitoyltransferase 1A [CPT1A] inhibitor; 4 µM) followed by the addition of BSA or palmitic acid conjugated with BSA (PA-BSA; 20 µM) for the next 2 h. Next, the cells were washed with DPBS containing Ca^2+^ and Mg^2+^ and placed on dry ice. Then, 300 µL of extraction solvent (80% methanol/water) cooled to −80 °C was added to each well, and the dishes were transferred to −80 °C for 15 min. Next, the cells were scraped and collected into the tubes, followed by washing the wells with an additional 100 µL of cooled methanol solution. The collected samples were vortexed briefly (~30 s) and sonicated on ice for 5 min to extract metabolites from cells. Two freeze–thaw cycles were used to accelerate the extraction of the cellular content. Next, all metabolite extracts were centrifuged at 4 °C and 20,000*g* for 20 min, and aliquots were retained in new 1.5 mL tubes. Finally, the solvent in each sample was evaporated in a vacuum concentrator (Labconco, Kansas City, MO, USA), and the samples were stored at −80 °C until preparation for the acyl-carnitine assay.

The instrumental analysis of methanol extracts was performed on a triple quadrupole QTRAP5500 liquid chromatography–tandem mass spectrometry (LC–MS/MS) system (AB Sciex, Framingham, MA, USA) equipped with a binary pump, a degasser and a column oven (Shimadzu, Kyoto, Japan) connected to a SIL-30AC autosampler. A Turbo V ion spray source operating in positive electrospray ionisation mode was used for ionisation. The extracted metabolites were chromatographically separated on a SeQuant ZIC-HILIC column (length 100 mm, internal diameter 2.1 mm, particle size 3.5 µm; Merck, Poznan, Poland) at a column temperature of 45 °C. Eluent A comprised 0.1% formic acid in water, and eluent B comprised 0.1% formic acid in acetonitrile (ACN). Gradient elution was performed using a 400 µL/min flow rate with the following program: 90% B for 2 min, a linear decrease to 75% B for 5 min, a linear decrease to 50% B for 3 min and a final decrease to 30% B for 1 min. Re-equilibration was attained at 90% B for 7 min. The total running time of the program was 18 min. Analytes were measured in multiple reaction monitoring (MRM) mode (each transition was performed with a dwell time of 10 ms; total scan time of 0.7 s for all 45 MRM transitions monitored). For quantification purposes, relevant ion transitions and the optimum collision energies (CE) were selected as follows: (1) carnitine: 162.2 → 85.1 (CE 25 eV); (2) acetylcarnitine: 204.1 → 85.0 (CE 24 eV); (3) propionylcarnitine: 218.1 → 85.0 (CE 24 eV); (4) butyrylcarnitine: 232.1 → 85.0 (CE 23 eV); (5) valerylcarnitine: 246.1 → 85.0 (CE 24 eV); (6) hexanoylcarnitine: 260.1 → 84.9 (CE 25 eV); (7) octanoylcarnitine: 288.2 → 85.1 (CE 25 eV); (8) decanoylcarnitine: 316.3 → 85.1 (CE 27 eV); (9) lauroylcarnitine: 344.2 → 85.1 (CE 28 eV); (10) myristoylcarnitine: 372.3 → 85.1 (CE 30 eV); (11) palmitoylcarnitine: 400.4 → 85.1 (CE 32 eV); (12) stearoylcarnitine: 428.4 → 85.1 (CE 32 eV); (13) oleoylcarnitine: 426.4 → 85.0 (CE 31 eV); (14) linoleoylcarnitine: 424.3 → 85.0 (CE 30 eV); (15) myristoylcarnitine-(*N*,*N*,*N*-trimethyl-d9; internal standard): 381.4 → 85.1 (CE 29 eV). The Analyst software (version 1.6) was used for data recording and processing.

### Analysis of free FAs

Primary LSECs were seeded at 150,000 per well in 96-well plates in EGM-2 medium and incubated overnight at 37 °C and 5% CO_2_. On the day of the experiment, the cells were washed twice with FAO buffer, followed by the addition of FAO buffer or BSA or PA-BSA (20 µM) in FAO buffer at a volume of 70 µL per well for 2 h of incubation at 37 °C. Each incubation buffer was also added to an empty well (without cells) for 2 h of incubation. Samples were collected and stored at −80 °C. The concentration of FAs was measured using a Free Fatty Acid Quantitation Kit according to the manufacturer’s protocol.

### Proteomic studies

#### Sample preparation for proteomic studies

Primary LSECs were seeded at 2 million per well in six-well plates in EGM-2 medium, incubated overnight at 37 °C and 5% CO_2_, washed twice with DPBS and scrapped. The material was frozen at −80 °C until analysed. The cells were lysed in lysis buffer (2.8 µL/mg sample, 4 °C) containing 7 M urea, 2 M thiourea, mass spectrometry-safe protease, phosphatase inhibitor cocktail (1:100) and 30 mM Tris–HCl (pH 8.0), sonicated on ice and centrifuged (16,000*g* for 15 min). The cell lysates were prepared for proteomic analysis according to Sitek et al. with slight modifications [28]. The protein concentration in the cell supernatant was assessed using the Bradford assay. Next, 10 µg of protein was dissolved in 258 µL of 50 mM ammonium bicarbonate (ABC). Reduction and alkylation were performed with 45 mM dithiothreitol in 50 mM ABC (15 min at 50 °C) and 100 mM iodoacetamide in 50 mM ABC (15 min at room temperature), respectively. Sequencing grade modified trypsin in a 1:50 w/w ratio was used to digest proteins (16 h at 37 °C). The digestion was quenched by adding 10 µL of formic acid. The samples were centrifuged (16,000*g* for 30 min), and the supernatant was lyophilised.

#### Mass spectrometry

Peptides were reconstituted in 0.1% trifluoroacetic acid with 2% ACN and subjected to LC–MS/MS analysis. Mass spectrometry was performed at the Mass Spectrometry Laboratory at the Institute of Biochemistry and Biophysics, Polish Academy of Sciences, Warsaw, Poland. Briefly, 10 μL of each sample was analysed using an LC–MS system comprising a UPLC chromatographer (nanoAcquity; Waters, MA) directly coupled to a QExactive mass spectrometer (Thermo Fisher Scientific). Peptides were trapped in a C18 pre-column (180 µm × 20 mm; Waters) using 0.1% FA in water as a mobile phase and then transferred to a nanoAcquity BEH C18 column (75 µm × 250 mm, 1.7 µm; Waters) using ACN gradient (0–35% ACN in 160 min) in the presence of 0.1% FA at a flow rate of 250 nL/min. The column temperature was set to 35 °C. The column outlet was coupled directly to the ion source of the mass spectrometer working in the regime of data-dependent MS to MS/MS switch (one of the top 12 data-dependent MS/MS scan methods). Full mass spectra were obtained from *m*/*z* 300 to 1650 with a resolution of 70,000. MS/MS spectra were acquired at a resolution of 17,500 and a maximum injection time of 60 ms for both MS1 and MS2. The automated gain control target was set to 1 × 10^6^ for MS1 and 2 × 10^5^ for MS2. The isolation window was set at 3 *m*/*z* and fixed first mass at 100 *m*/*z*. Dynamic exclusion was set to 30 s. A blank run preceded each analysis to ensure the absence of cross-contamination from previous samples.

#### Identification of proteins

The acquired MS/MS data were preprocessed with Mascot Distiller software (v.2.6.2; MatrixScience, London, UK), and a search was performed with the Mascot Search Engine (Mascot Server 2.5; MatrixScience) against the human and murine proteins deposited in the SwissProt database. The peptide and fragment mass tolerance settings were established separately for individual LC–MS/MS runs after a measured mass recalibration to reduce mass errors, as previously described [29]. The other search parameters were as follows: enzyme, trypsin; missed cleavages, 1; fixed modifications, carbamidomethyl (C); variable modifications, oxidation (M); instrument, HCD. A statistical assessment of the confidence of peptide assignments was based on the target/decoy database search strategy, and the Mascot score threshold was adjusted to a 1% false positive rate.

#### Analysis of the proteomic data

The exponentially modified protein abundance index (emPAI) [30] was calculated using the in-built tool of Mascot Search Engine (Mascot Server 2.5; MatrixScience) to detect and relatively quantify proteins. The emPAI values were normalised to the total emPAI value and compared as a percentage of the overall composition [31]. Furthermore, to consider the relative content of specific proteins of interest in the cell proteome, we presented in the Additional file 1: Supplementary Table 1 the obtained results as the relative abundance (emPAI%) and also in relation to the glyceraldehyde-3-phosphate dehydrogenase (GAPDH) content.

### Analysis of catalase activity

To analyse catalase (CAT) activity in LSECs in reference to HAECs, MDA-MB-231 cells and hLMVECs, cells were plated into six-well plates to reach confluence. Next, the cells were washed twice with DPBS. Then, 200 µL of DPBS containing 0.1% Triton X-100 and a protein inhibitor cocktail (1:100) was added before the cells were collected by scrapping and frozen at −80 °C. CAT activity was determined spectrophotometrically using the method of Koroliuk et al. [32]. Briefly, 10 µL of the sample was mixed with 200 µL of 0.03% hydrogen peroxide and incubated for 10 min at 37 °C. The reaction was terminated by adding 100 µL of 4% ammonium molybdate, which forms a yellow complex with hydrogen peroxide that can be measured spectrophotometrically at 410 nm. One unit of CAT activity (1 Kat) was defined as the amount of enzyme required to decompose 1 µmol of hydrogen peroxide per min per mg of protein.

### Raman imaging

The deuterium labelled PA (d-PA) for the experiments was saponified using 100 mM NaOH and conjugated with FA-free BSA (in DPBS) at a 6:1 molar ratio, producing d-PA-BSA at d-PA concentration of 1 mM and BSA concentration of 0.167 mM. LSECs were incubated with PA-BSA (20 µM; Agilent) or d-PA-BSA (20 µM) for 2 h, fixed in 2.5% glutaraldehyde for 10 min and imaged using Raman spectroscopy. A WITec Confocal Raman Imaging System (alpha300; WITec, Ulm, Germany) equipped with a UHTS 300 spectrograph (600 grooves/mm grating, 3 cm^−1^ resolution) and an Andor CCD detector (DU401A-BV-352) was used for Raman imaging. Raman spectra of LSECs were acquired using a 40× water immersive objective (Zeiss Fluor, NA of 1.0), with a laser wavelength of 532 nm, a maximum laser power of approximately 30 mW at the sample position and an exposure time of 0.3 s per spectrum. The nominal lateral resolution for our setup was 0.32 μm, and a sampling density of 0.50 in *x*/*y* was used. Details of this experimental setup has been previously reported [33, 34]. The data matrices were analysed using WITec Project Five 5.1 software, with background subtraction performed using a third-degree polynomial and automatic removal of cosmic rays. The heterogeneity of lipid droplets (LDs) in LSECs was studied by extracting the Raman spectra from the centre of each LD and then averaging them.

### Statistical analysis

Statistical analyses were performed using OriginPro 2022b software (OriginLab Corporation, Northampton, MA). The results were expressed as the mean ± standard deviation (SD) or the mean ± standard error of the mean (SEM), depending on the type of analysis. The data were checked for normality and homogeneity of variances. The data were compared using one-way analyses of variance (ANOVA) with a Bonferroni test or Student’s *t*-test. The numbers of independent experiments, technical replicates and *p*-values are provided in the legends.

## Results

### LSECs have a proteome profile related to mitochondrial energy metabolism

**Fig. 1:**
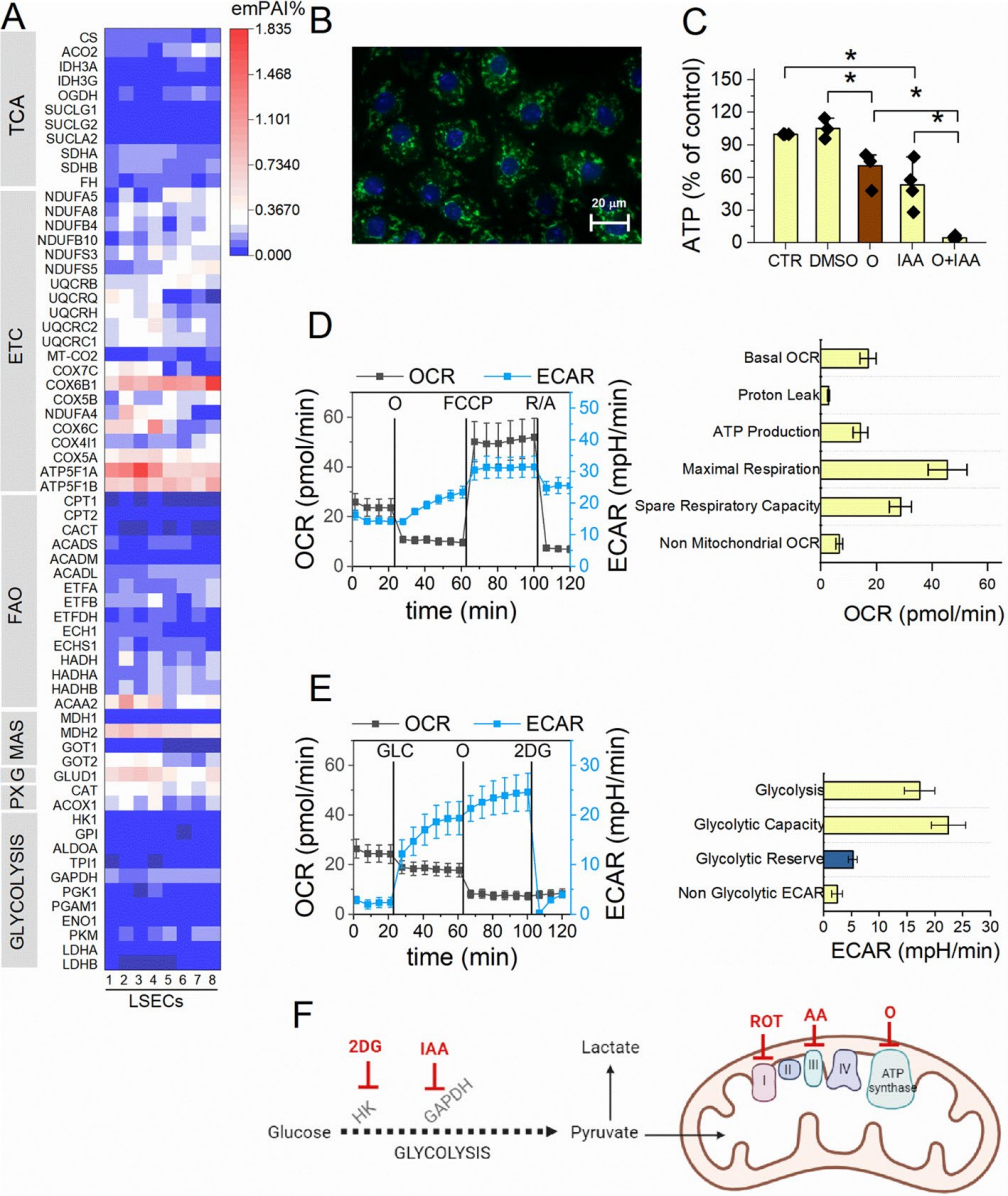
Assessment of oxidative phosphorylation and glycolysis in LSECs. **A** A heat map of the relative abundance (emPAI%) of selected proteins identified in LSECs related to the TCA cycle, ETC, FAO in mitochondria, MAS, glutamine metabolism (G), peroxisomes and FAO in peroxisomes (PX) and glycolysis. The data are derived from eight replicates. **B** A representative image of LSEC mitochondria. Mitochondria (green) were labelled with MitoTracker Green FM, and nuclei (blue) were labelled with Hoechst 33342. **C** Changes in ATP concentration in LSECs treated for 1 h with DMSO (as a vehicle), oligomycin (O; 1 µg/mL) or IAA (20 µM). Data are shown as means ± SEMs from four independent experiments (with three replicates per experiment). Significance was tested using one-way ANOVA (**A**, **B**); *p < 0.05. **D** The OCR (grey) and ECAR (blue) in LSECs measured using the MST; the mitochondrial function parameters were calculated from the kinetic data. Data are shown as means ± SEMs from three independent experiments (with 5–6 replicates per experiment). **E** The ECAR (blue) and OCR (grey) in LSECs measured using the GST; the glycolytic function parameters were calculated from the kinetic data. Data are shown as means ± SEMs from three independent experiments (with 5–6 replicates per experiment). **F** Schematic presentation of glycolysis and oxidative phosphorylation with molecular targets for applied inhibitors (HK, hexokinase). The image was created using BioRender.com

To explore the repertoire of enzymes involved in energy metabolism in LSECs, we analysed their proteome using an untargeted LC–MS/MS-based method. We confirmed a relatively high abundance of proteins involved in the electron transport chain (ETC; particularly complexes IV and V), glutamine metabolism, malate–aspartate shuttle (MAS), and peroxisome function, and a relatively low abundance of proteins involved in glycolysis, with GAPDH having the highest content (emPAI% 0.136 ± 0.034). Figure 1A presents results for selected proteins involved in energy metabolism pathways to show their relative abundances; more detailed data are shown in Additional file 1: Fig. S2 and Supplementary Table 1. All ETC complexes (Additional file 1: Fig. S2B–F), as well as mitochondrial (Additional file 1: Fig. S2G, H) and peroxisomal (Additional file 1: Fig. S2I) proteins involved in FAO, were highly expressed in LSECs.

LSECs contained relatively high contents of enzymes directly involved in the mitochondrial β-oxidation pathway: acyl-coenzyme A (CoA) dehydrogenases (ACADs; several enzymes responsible for the first step of β-oxidation, specific for different types of FAs), enoyl-CoA hydratase short chain 1 (ECHS1), hydroxyacyl-CoA dehydrogenase (HADH; Additional file 1: Fig. S2G) and especially high β-ketoacyl-CoA thiolase (ACAA2; Additional file 1: Fig. S2H). Additionally, there were also relatively high contents of electron transfer flavoprotein (ETF) subunits alpha/beta (ETFA/B), representing a primary source of reducing power for the ETC from both FA and amino acid oxidation, and electron transfer flavoprotein-ubiquinone oxidoreductase (ETFDH; Additional file 1: Fig. S2G), which participates in electron transfer from various mitochondrial dehydrogenases to ubiquinone in the ETC [35, 36]. Therefore, the ETF became known as a central hub in mitochondrial redox metabolism.

Furthermore, LSECs contained relatively high levels of peroxisomal proteins, especially acyl-CoA oxidase 1 (ACOX1) and CAT (Additional file 1: Fig. S2I). Particularly high levels of mitochondrial components of the second important player in cellular redox metabolism, the MAS (malate dehydrogenase 2 [MDH2] and glutamic-oxaloacetic transaminase 2 [GOT2]) were observed in LSECs (Additional file 1: Fig. S2L), suggesting the importance of NAD^+^/NADH turnover and redox reactions. LSECs also showed relatively high abundances of enzymes involved in pyruvate (pyruvate dehydrogenase [PDH] and pyruvate carboxylase [PC]; Additional file 1: Fig. S2J) and glutamine (mitochondrial glutamate dehydrogenase 1 [GLUD1] and GOT2; Additional file 1: Fig. S2K) utilisation for energy metabolism. The gene ontology (GO)-enrichment analysis revealed that the most enriched proteins in LSECs were related to mitochondria, generation of precursor metabolites and energy and electron transfer activity (Additional file 1: Fig. S3). Altogether, the proteomic data revealed that LSECs were highly equipped with a proteome that allows efficient FAO and oxidative phosphorylation with a relatively low content of glycolytic enzymes (Figs. 1A, Additional file 1: Fig. S2A). This finding supports the notion of the higher dependence of LSECs on oxidative phosphorylation.

### LSECs rely primarily on oxidative phosphorylation for ATP production

Primary LSECs have tubular and thick mitochondria (Fig. 1B). To investigate the contribution of mitochondrial respiration and glycolysis to ATP production in primary LSECs, we measured the intracellular ATP concentration and performed mitochondrial (MST) and glycolysis (GST) stress tests. When added individually, oligomycin (a mitochondrial ATP synthase inhibitor) or IAA (a GAPDH inhibitor) reduced the ATP concentration, but when added together, they completely blocked ATP production (Fig. 1C, F). For comparison, another type of EC (human aortic ECs [HAECs]) with a distinct proteome profile relied primarily on glycolysis for ATP production (Additional file 1: Fig. S4 and Supplementary Table 2).

**Fig. 2:**
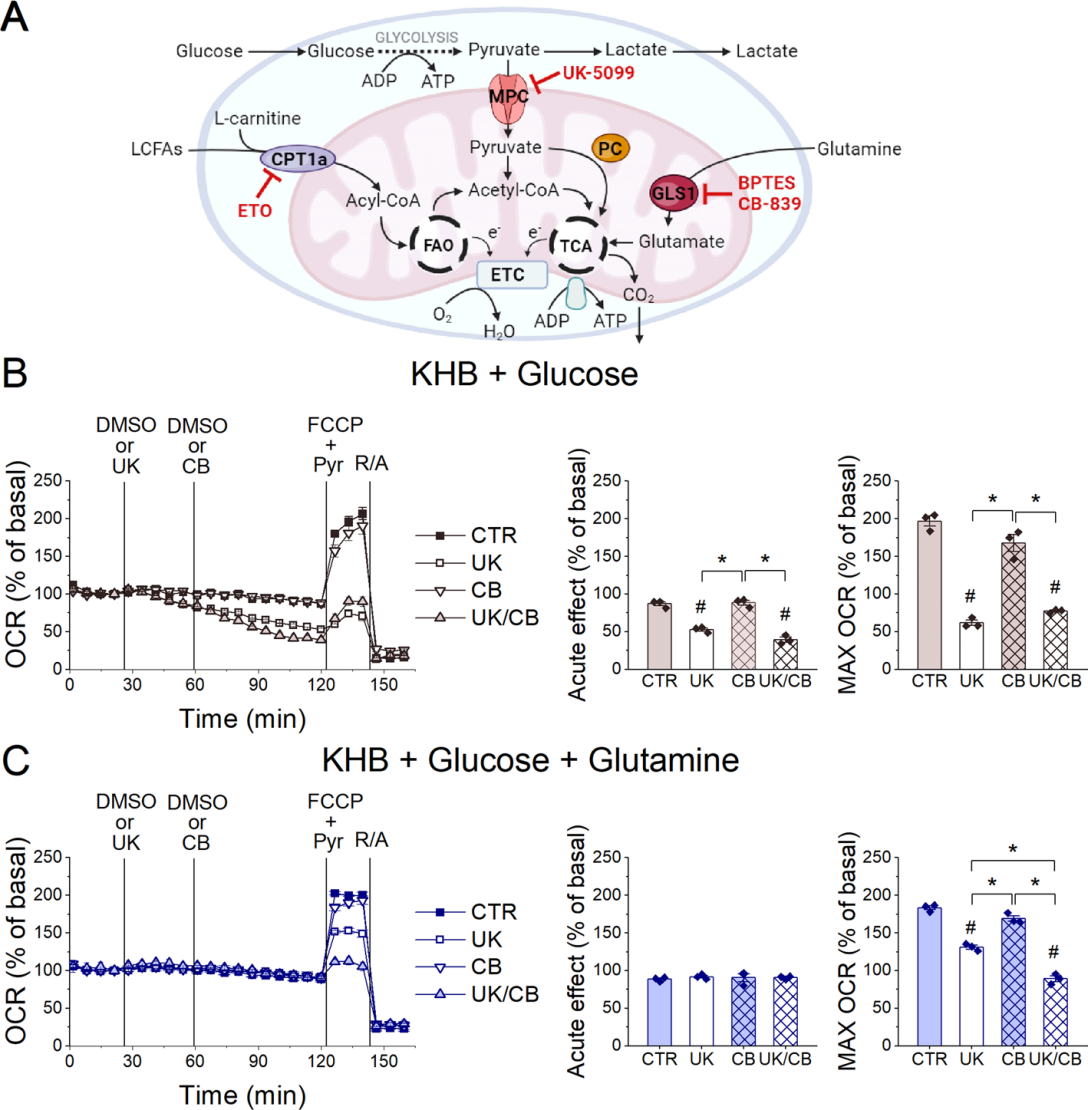
Glucose and glutamine utilisation by LSECs. **A** A schematic representation of the metabolic pathways that supply oxidative phosphorylation with molecular targets for their inhibitors. The image was created with BioRender.com. **B**, **C** The OCR measured in LSECs in KHB supplemented with glucose only (5.5 mM; **B**) or glucose with glutamine (2 mM; **C**) and subsequently treated with DMSO (as a vehicle), UK-5099 (10 µM), CB-839 (5 µM) or UK/CB, followed by pyruvate (1 mM) with FCCP (2 µM) and R/A (0.5/0.5 µM); acute effects before the addition of FCCP and the maximal OCR were calculated from the kinetic data. Data are shown as means ± SEMs from three independent experiments (with three to four replicates per experiment). Significance was tested using one-way ANOVA (**B**, **C**); *p < 0.05; ^#^*p* < 0.05 compared with control

**Fig. 3:**
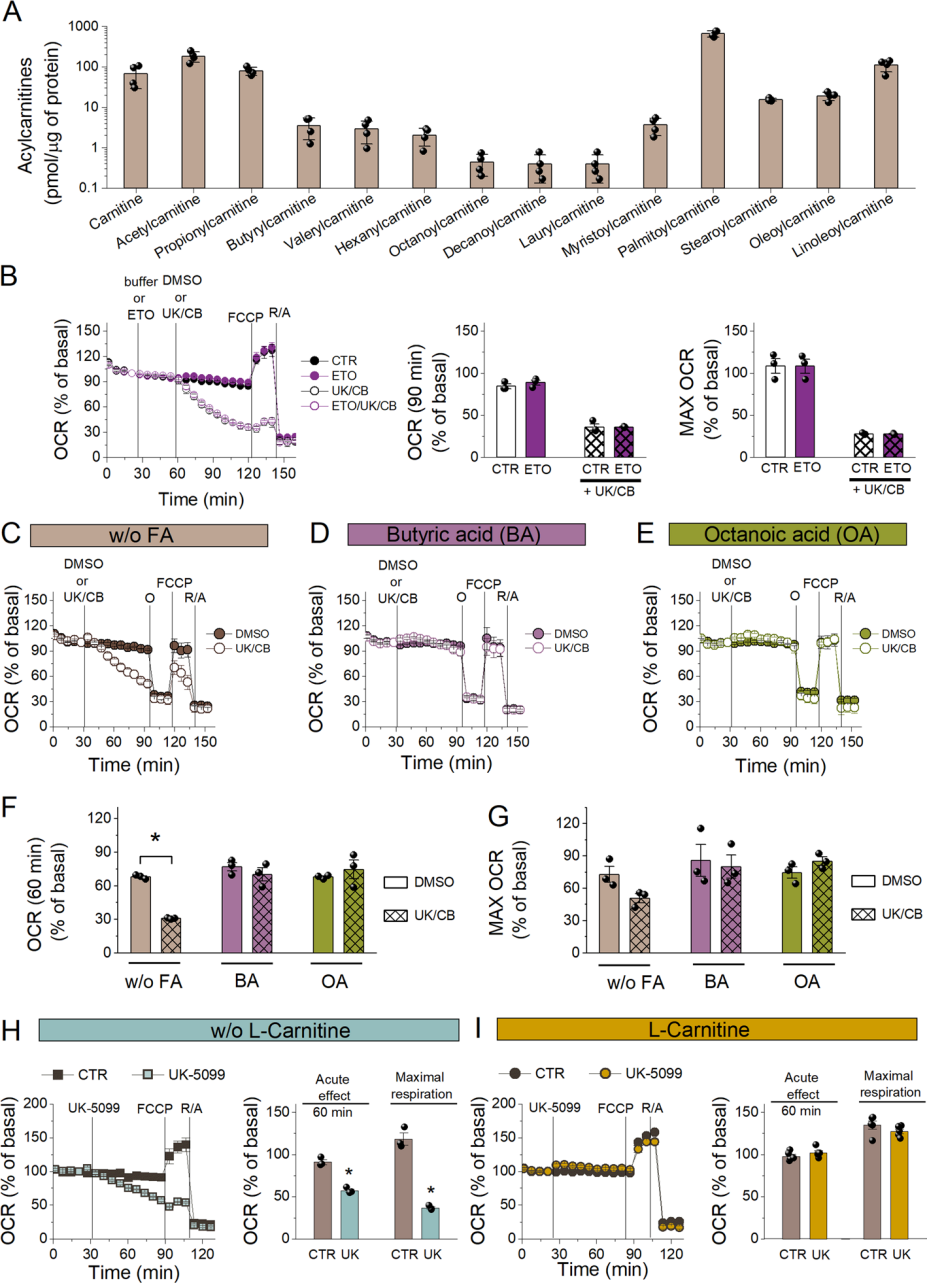
Endogenous fatty acid utilisation by LSECs and the importance of l-carnitine. **A** Acylcarnitine levels in LSECs incubated for 2 h in KHB supplemented with glucose (2.5 mM) and l-carnitine (50 µM). Data are shown as means ± SDs from four independent experiments. **B** The OCR measured in LSECs in KHB supplemented with glucose (2.5 mM), untreated or treated with ETO (4 µM), followed by DMSO or UK-5099 (10 µM) and CB-839 (5 µM), then FCCP (2 µM) and R/A (0.5/0.5 µM). Changes in basal respiration (90 min after ETO addition) and the maximal OCR were calculated from the kinetic data. Data are shown as means ± SEMs from three independent experiments (with three to four replicates per experiment). **C**–**G** The OCR measured in LSECs in KHB supplemented with glucose (2.5 mM) without exogenous FAs (w/oFA; **C**), with 100 µM BA (**D**) or 100 µM OA (**E**) added to the incubation buffer just before the start of measurements. After the four basal measurements, DMSO or UK-5099 (10 µM) and CB-839 (5 µM) were added for 60 min, followed by FCCP (2 µM) and R/A (0.5/0.5 µM). Changes in basal respiration (60 min after UK/CB addition; **F**) and the maximal OCR (G) were calculated from the kinetic data presented in **C**–**E**. Data are shown as means ± SEMs from three independent experiments (with three to four replicates per experiment). **H**, **I** The OCR measured in LSECs in KHB supplemented with glucose only (2.5 mM; **H**) or with glucose and l-carnitine (500 µM; I), followed by UK-5099 (10 µM), FCCP (2 µM) and R/A (0.5/0.5 µM). Changes in basal respiration (60 min after UK-5099 addition) and the maximal OCR were calculated from the kinetic data. Data are shown as means ± SEMs from three independent experiments (with three to four replicates per experiment)

In the MST—performed in unbuffered Dulbecco’s modified Eagle’s medium (DMEM) supplemented with glucose, glutamine, and pyruvate—oligomycin induced a rapid decrease in the OCR, followed by a substantial increase in response to FCCP (an uncoupler) and finally a substantial decrease after treatment with R/A (Fig. 1D), indicating highly functional mitochondria. Simultaneous ECAR measurements demonstrated that glycolysis was gradually activated in LSECs after ATP synthase inhibition by oligomycin. A significant decrease in ECAR after adding R/A confirmed a substantial contribution of CO_2_-derived* HCO*_3_^-^ to the observed extracellular acidification. Interestingly, shortly after cell isolation (2 h), the ECAR resulted almost entirely from * HCO*_3_^-^, confirmed by a sharp decrease in ECAR after oligomycin treatment (Additional file 1: Fig. S5A). Importantly, the shape of the ECAR response to mitochondrial modulators in LSECs resembled the shape of the OCR, confirming a strong dependence of LSECs on mitochondrial respiration (Additional file 1: Fig. S5A). Collectively, (1) the relatively high abundance of proteins related to mitochondrial respiration (Fig. 1A), (2) the oligomycin effect on ATP concentration (Fig. 1C) and (3) the MST profile (Fig. 1D) all suggest that LSECs efficiently used oxidative phosphorylation for ATP production.

In the GST, adding glucose (GLC; 10 mM) to unbuffered DMEM supplemented with glutamine (2 mM) induced a substantial increase in the ECAR with a slight simultaneous decrease in the OCR in LSECs (Fig. 1E). However, changes in the ECAR were gradual, not rapid (Fig. 1E). Moreover, subsequent ATP synthase inhibition with oligomycin induced a further increase in the ECAR, indicating a substantial glycolytic reserve (Fig. 1E). Applying 2-deoxy-d-glucose (2DG, 50 mM; Fig. 1E) at the end of the measurements confirmed that the observed extracellular acidification resulted from glycolysis. Shortly after cell isolation (2 h), extracellular acidification resulted almost exclusively from mitochondrial respiration (Additional file 1: Fig. S5B), suggesting a transfer of glucose-derived pyruvate directly to mitochondria. Figure 1F demonstrates the schematic presentation of molecular targets for applied metabolic inhibitors. Altogether, (1) the proteome profile (Fig. 1A), (2) the significant decrease in intracellular ATP level after oligomycin (Fig. 1C) and (3) the OCR and ECAR profiles in the MST and GST assays support the conclusion that glycolysis is not a predominant ATP production pathway in LSECs.

### Glucose-derived pyruvate and glutamine are important metabolic fuels for oxidative phosphorylation in LSECs

In the mitochondrial fuel flexibility test (MFFT), designed to assess substrate preference, the OCR in LSECs was almost unaffected after treatment with 2-cyano-3-(1-phenyl-1*H*-indol-3-yl)-2-propenoic acid (UK-5099, a mitochondrial pyruvate carrier [MPC] inhibitor, 2 µM), bis-2-(5-phenylacetamido-1,3,4-thiadiazol-2-yl)ethyl sulfide (BPTES, a glutaminase 1 [GLS1] inhibitor, 3 µM) and ETO (4 µM), applied in different combinations (Fig. 2A; Additional file 1: Fig. S6). These data show that even under combined treatment with UK-5099, BPTES and ETO, mitochondrial respiration was unaffected in LSECs incubated in DMEM supplemented with glucose (5.5 mM), glutamine (2 mM) and pyruvate (1 mM), confirming their high metabolic plasticity.

Notably, the utilisation of glucose-derived pyruvate and glutamine for energy metabolism in LSECs was examined in KHB supplemented with glucose or glucose with glutamine to avoid the effects of other DMEM components that are potential metabolic fuels. Figure 2B shows that MPC inhibition by UK-5099 (10 µM) induced a gradual but substantial decrease in the basal OCR and an even stronger decrease in the maximal OCR in KHB supplemented only with glucose (5.5 mM). However, UK-5099 did not affect the basal OCR when glutamine (2 mM) was also present in the KHB (Fig. 2C); only a decrease in the maximal OCR was observed. This indicates that glucose-derived pyruvate was an important fuel for energy metabolism, either as a source of acetyl-CoA oxidised in the tricarboxylic acid (TCA) cycle or as an anaplerotic substrate when converted into oxaloacetate by PC, while glutamine supported mitochondrial respiration.

The GLS1 inhibitor, *N*-(5-[4-[6-[[2-[3-(trifluoromethoxy)phenyl]acetyl]amino]-3-pyridazinyl]butyl]-1,3,4-thiadiazol-2-yl)-2-pyridineacetamide (CB-839, 5 µM), only inhibited maximal respiration in LSECs after prior treatment with UK-5099 (Fig. 2C). A high level of mitochondrial GLUD1 (Fig. 1A, Additional file 1: Fig. S2K) and the dynamics of OCR changes in response to energy stress (rapid with and slow without glutamine; Fig. 2), confirmed the importance of glutamine as an anaplerotic substrate for the TCA cycle, consistent with the results of Spolarics et al. on glutamine utilisation by LSECs [19].

### SCFAs and MCFAs are substrates for oxidative phosphorylation in LSECs

To investigate the utilisation of endogenous FAs for energy metabolism in LSECs, we measured the acylcarnitine profile in LSECs incubated for 2 h in KHB supplemented with 2.5 mM glucose and 50 µM l-carnitine (Fig. 3A, Additional file 1: Supplementary Table 3). We identified palmitoylcarnitine as the most abundant and SCFA- and MCFA-carnitines (particularly octanoylcarnitine, decanoylcarnitine and laurylcarnitine) as the least abundant. The accumulation of palmitoylcarnitine confirmed that palmitic acid (PA) was available for oxidation in LSECs. However, ETO (4 µM), expected to inhibit oxygen consumption related to PA oxidation [37], did not affect the OCR in LSECs under basal conditions (90 min after addition) or energy stress (maximal respiration after FCCP) and did not enhance the action of UK-5099 and CB-839 (Fig. 3B). Higher ETO concentrations (10 and 40 µM; data not shown) and other known CPT1A inhibitors (perhexiline [PER; 5 µM] and oxfenicine [1 mM]; Additional file 1: Fig. S7) were also ineffective, although an uncoupling effect was observed with PER. These results indicate that long-chain FAs (LCFAs) were not directly utilised in mitochondria, unlike SCFAs and MCFAs. The low abundance of SCFA- and MCFA-carnitines (Fig. 3A) suggested that SCFAs and MCFAs were preferentially used for energy metabolism in LSECs.

To examine the utilisation of SCFAs and MCFAs for oxidative phosphorylation, we investigated the effects of exogenously added butyric acid (BA; a representative SCFA; C4:0) and octanoic acid (OA; a representative MCFA; C8:0) on mitochondrial function in LSECs. LSECs were washed with KHB containing 2.5 mM glucose but not l-carnitine and incubated for 1 h, followed by adding the assay buffer without exogenous FAs or supplemented with BA (100 µM) or OA (100 µM). After measuring basal respiration, UK-5099 and CB-839 were added, and the OCR was measured for another 60 min, followed by the MST. In LSECs supplemented only with glucose, combining UK-5099 and CB-839 (UK/CB) reduced the basal OCR (Fig. 3C), while supplementation with BA or OA abolished the inhibitory effect of UK/CB (Fig. 3D, E). In addition, basal respiration and the maximal OCR were unaffected by UK/CB in the presence of BA or OA (Fig. 3D–G). These data demonstrate that LSECs could efficiently utilise exogenously added SCFAs and MCFAs to support mitochondrial respiration.

Interestingly, while UK-5099 (10 µM) efficiently reduced basal and maximal respiration in LSECs incubated in KHB supplemented with glucose (2.5 mM) only (Fig. 3H), its effect on basal and maximal respiration was abolished in the presence of l-carnitine (500 µM; Fig. 3I), indicating the importance of l-carnitine for mitochondrial respiration when utilising endogenous substrates. In addition to participating in the transport of LCFAs from the cytoplasm to mitochondria, l-carnitine also enables the transfer of SCFAs and MCFAs formed in peroxisomes to mitochondria for further oxidation [38].

Altogether, (1) the acylcarnitine profile of LSECs, (2) the lack of an inhibitory effect by ETO and (3) the efficient mitochondrial respiration based on SCFAs (BA) and MCFAs (OA) demonstrate that LSECs prefer SCFAs and MCFAs over LCFAs for oxidation. In addition, the effect of l-carnitine on mitochondrial respiration without supplementation with exogenous FAs and with the blocking of pyruvate transport to mitochondria, resulting in the blocking of pyruvate-derived acetyl-CoA utilisation in the TCA cycle, strongly suggests that endogenous LCFAs are first shortened in peroxisomes and then delivered to mitochondria as SCFAs and MCFAs.

### PA accumulates in lipid droplets

**Fig. 4:**
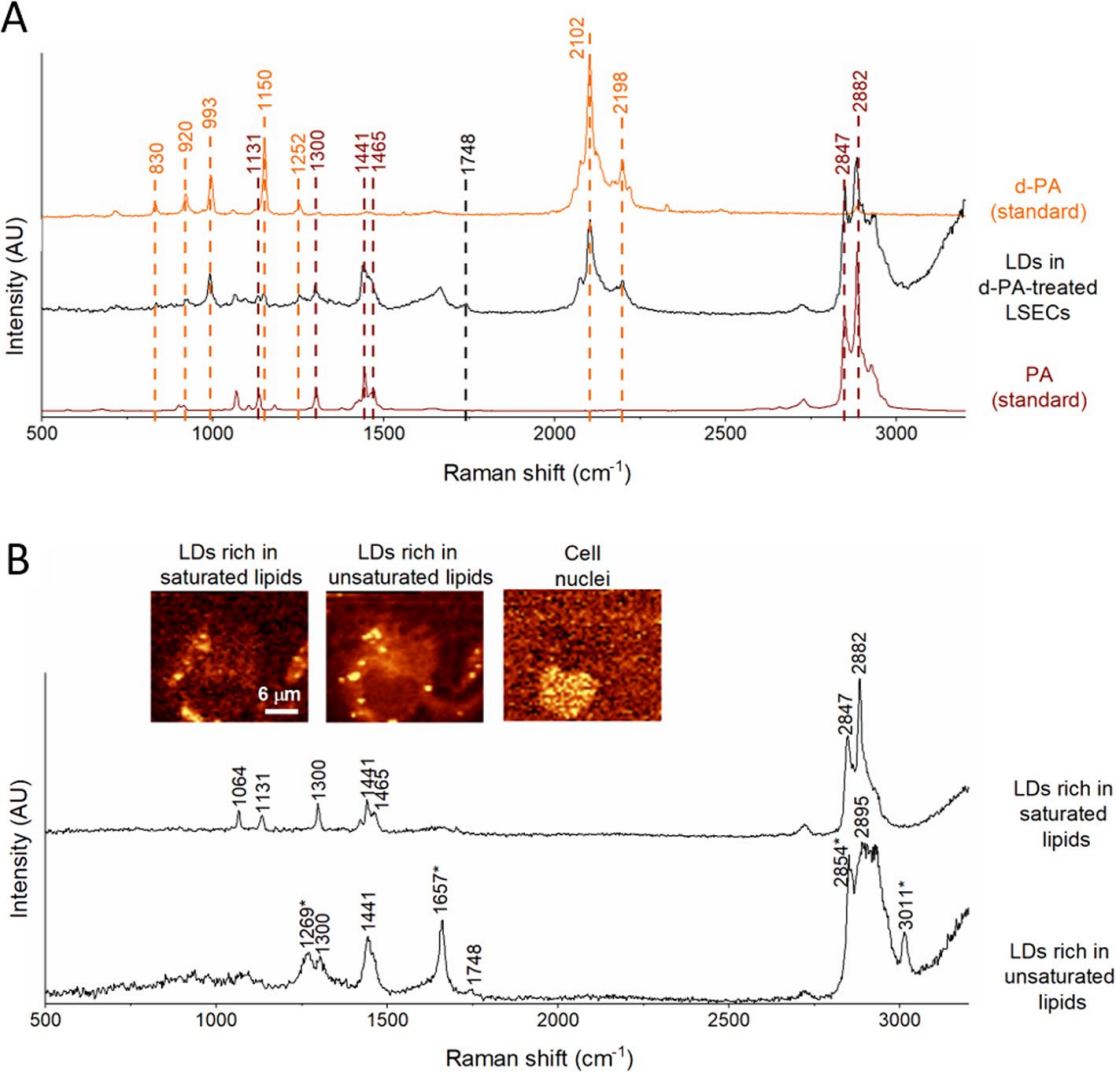
Palmitic acid is taken up and stored in LDs in LSECs. **A** Representative Raman spectra of LDs extracted from LSECs (black) compared with the Raman spectrum of d-PA (orange) and PA (brown). The Raman spectra of LDs in LSECs incubated with d-PA contain several Raman bands (orange), indicating the presence of deuterated lipids inside the LDs. **B** Representative Raman images of LSECs showing the distribution of DNA and RNA (778–808 cm^−1^), saturated lipids (2866–2899 cm^−1^) or unsaturated lipids (2995–3030 cm^−1^) after a 2 h incubation with PA. In the Raman images, colour intensity increases with band intensity. LSECs contain LDs rich in saturated and unsaturated lipids (Raman bands corresponding to vibrations of unsaturated moieties are denoted with *)

**Fig. 5:**
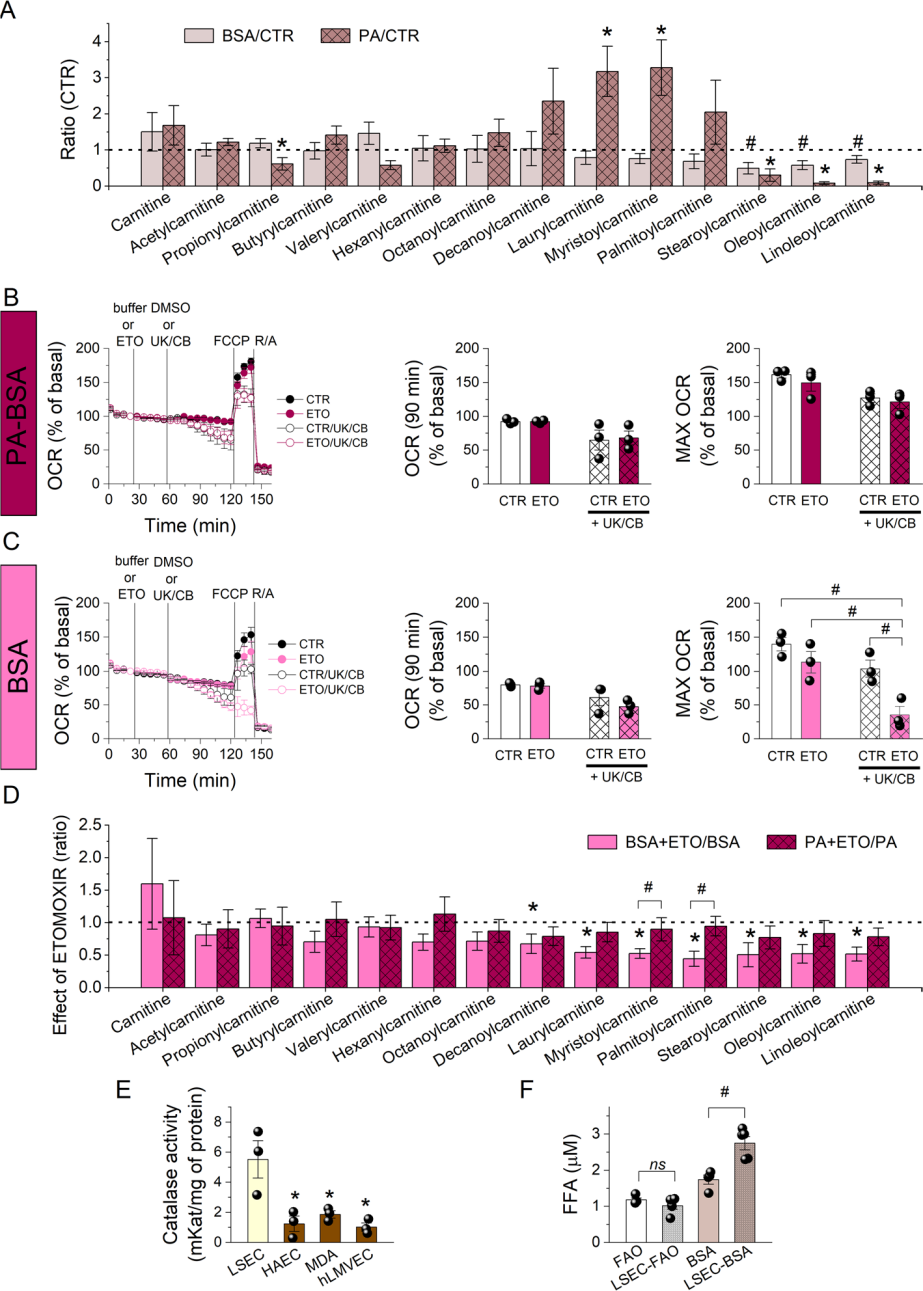
Palmitic acid fate in LSECs. **A** The relative levels of acylcarnitines in LSECs incubated for 2 h with BSA (3.3 µM) or PA-BSA (20/3.3 µM) in KHB supplemented with glucose (2.5 mM) and l-carnitine (50 µM) normalised to the controls (without BSA and PA-BSA). Data are shown as means ± SDs from four independent experiments; *^,#^*p* < 0.05. **B** The OCR measured in LSECs in KHB supplemented with glucose (2.5 mM), l-carnitine (50 µM) and PA-BSA (20 µM/3.3 µM), untreated or treated with ETO (4 µM) and followed by DMSO or UK-5099 (10 µM) and CB-839 (5 µM), then FCCP (5 µM) and R/A (1/1 µM). Changes in basal respiration (90 min after ETO addition) and the maximal OCR were calculated from the kinetic data. Data are shown as means ± SEMs from three independent experiments (with three to four replicates per experiment). **C** The OCR measured in LSECs in KHB supplemented with glucose (2.5 mM), l-carnitine (50 µM) and BSA (3.3 µM), untreated or treated with ETO (4 µM) and followed by DMSO or UK-5099 (10 µM) and CB-839 (5 µM), then FCCP (5 µM) and R/A (1/1 µM). Changes in basal respiration (90 min after ETO addition) and the maximal OCR were calculated from the kinetic data. Data are shown as means ± SEMs from three independent experiments (with three to four replicates per experiment); ^#^*p* < 0.05. **D** Relative levels of acylcarnitines in LSECs pre-incubated for 15 min with ETO (4 µM) and then incubated for 2 h with BSA (3.3 µM) or PA-BSA (20/3.3 µM) in KHB supplemented with glucose (2.5 mM) and l-carnitine (50 µM) normalised to the controls without ETO. Data are shown as means ± SDs from four independent experiments; *p < 0.05 compared with the control; ^#^*p* < 0.05. **E** The CAT activity of LSECs, HAECs, MDA-MB-231 cells and hLMVECs. Data are shown as means ± SDs from three independent experiments. **F** Free FA concentration in FAO buffer (KHB supplemented with glucose [2.5 mM] and l-carnitine [50 µM]) with or without BSA (3.3 µM) after 2 h of incubation with or without LSECs. Data are shown as means ± SDs from five independent experiments; ^#^*p* < 0.05

To investigate whether LCFA oxidation is important for LSEC energy metabolism, we characterised the trafficking of PA (a representative LCFA; C16:0), the levels of acylcarnitines and the use of PA for mitochondrial respiration. Based on the presence of spectral bands characteristic of PA, Raman spectroscopy imaging confirmed that PA was taken up by LSECs and stored in LDs (Fig. 4A). LDs formed two distinct populations based on their inclusion of unsaturated or saturated lipids (Fig. 4B). LSECs incubated with PA-BSA (6:1) contained more saturated LDs than those incubated with BSA alone. The utilisation of deuterated PA (d-PA) further confirmed, based on characteristic bands, the trafficking of d-PA to LDs to be stored in an esterified form (Fig. 4A). Furthermore, the acylcarnitine profile of LSECs incubated for 2 h with PA-BSA in KHB supplemented with glucose (2.5 mM) and l-carnitine (50 µM) clearly showed enrichment in laurylcarnitine and myristoylcarnitine and depletion in stearoylcarnitine, oleoylcarnitine and linoleoylcarnitine (Fig. 5A; Additional file 1: Supplementary Table 1). The enrichment in acylcarnitines shorter than palmitoylcarnitine indicates the shortening of PA inside LSECs, while the depletion in stearoylcarnitine, oleoylcarnitine and linoleoylcarnitine indicates the lack of preference for using stearic, oleic and linoleic acids due to the increased availability of PA. Altogether, the data presented in Figs. 4 and 5A confirm that (1) exogenous PA was taken up by cells and transformed to palmitoylcarnitine and (2) PA was oxidised inside LSECs, generating shorter FAs.

We measured oxygen consumption to investigate whether ETO could inhibit mitochondrial respiration in the presence of exogenously added PA (PA-BSA; 20 µM). Figure 5B shows that ETO (4 µM) did not affect the OCR in LSECs under basal conditions or energy stress (maximum respiration after FCCP) and did not enhance the action of UK-5099 or CB-839. ETO also did not affect the acylcarnitine profile in the presence of exogenously added PA (Fig. 5D), indicating that the formation of acylcarnitines was independent of CPT1A. Additional file 1: Fig. S2G shows relatively low abundance of CPT1A in the LSEC proteome (identified in only two of eight samples, with its analytical signal below the detection threshold in the other six samples); it was lower than in HAECs and breast cancer MCF-7 cells, as confirmed by western blot analysis (Additional file 1: Fig. S8). Furthermore, LSECs had high levels of peroxisomal β-oxidation enzymes, particularly ACOX1 (Additional file 1: Fig. S2I), but also a high CAT level (Additional file 1: Fig. S2I) and activity (Fig. 5E). These data suggest that LCFAs, stored in LDs or added exogenously, are shortened to MCFAs and SCFAs by oxidation in peroxisomes under increased energy demands before being transported into mitochondria.

Surprisingly, incubating LSECs with BSA resulted in a slight but significant depletion of stearoylcarnitine, oleoylcarnitine and linoleoylcarnitine, indicating increased consumption of LCFAs and suggesting that BSA was not neutral to LSECs but induced a decrease in FA availability in them (Fig. 5A). To evaluate whether BSA could induce endogenous FA efflux, we measured the concentration of free FAs in the BSA-containing incubation buffer. The free FA levels in the incubation buffer (2 h) were higher in the presence of LSECs (Fig. 5F). Furthermore, only in the BSA condition, ETO decreased the maximal OCR after FCCP addition (Fig. 5C). Analysis of the acylcarnitine profile demonstrated that pretreatment of LSECs with ETO (4 µM) was ineffective with PA-BSA but resulted in a substantial depletion of LCFA-carnitines with BSA alone (Fig. 5D). This difference could be caused by increased SCFA and MCFA efflux in the assay buffer with BSA, accompanied by accelerated utilisation of LCFAs not only in peroxisomes but also directly in mitochondria, enabled by residual CPT1A. Cui et al. recently described a similar effect in the presence of BSA in a serum-free incubation medium and associated it with an efflux of lipophagy-derived FAs [39]; the issues related to using BSA as a carrier for free FAs were widely reviewed by Alsabeeh et al. [40]. In summary, these data demonstrate that extracellular BSA causes a partial efflux of FAs from LSECs, decreasing their availability in cells.

## Discussion

It is widely accepted that ECs rely mainly on glycolysis for ATP generation [11, 12], using their mitochondria more in regulating intracellular calcium, reactive oxygen species (ROS)-dependent signalling and redox regulation [15, 16]. Eelen et al. extensively discussed the various benefits of the dependence of most types of ECs on glycolysis [41]. However, the high heterogeneity of ECs reflects their morphological and functional adaptations to meet the unique demands of the underlying tissue and their specific functions. This study demonstrates that LSECs rely on oxidative phosphorylation for ATP production, efficiently fuelled by glucose-derived pyruvate, SCFAs, MCFAs and glutamine as an anaplerotic substrate, confirming their high metabolic plasticity and emphasising their unique metabolic features compared with other types of ECs, which rely mainly on glycolysis. In addition, our results suggest that LCFAs are not directly oxidised in mitochondria, underscoring the possibly important role of peroxisomes in degrading these FAs in LSECs.

### Oxidative phosphorylation prevails over glycolysis for ATP production in LSECs

In this study, we showed via proteomics and functional assays that primary LSECs relied more on oxidative phosphorylation than glycolysis to produce ATP. These results are consistent with the conclusions of Spolarics et al. [19] and Dudek et al. [20]. The strong dependence of LSECs on oxidative phosphorylation is surprising, and its importance for liver homeostasis remains incompletely understood.

This unique energy metabolism in LSECs may be related to their high endocytic capacity and scavenging function, which is distinct from other types of ECs [42]. Indeed, the high-energy catabolic demands supplied by oxidative phosphorylation in LSECs may be required to maintain their scavenging and antigen-cross-presentation function; interestingly, antigen-cross-presentation also requires low-level glycolysis for anabolic processes [20]. Notably, LSECs can internalise many viruses through endocytosis and then degrade viral particles in lysosomes to activate antiviral defence [42–46], with peroxisomes potentially supporting this response. Indeed, in addition to being responsible for the metabolism of complex lipids and the detoxification of reactive species, these organelles also regulate various immune functions [47]. Dixit et al. demonstrated that peroxisomes function together with mitochondria to amplify and stabilise the antiviral response by inducing a rapid interferon-dependent response to viral infection and activating a delayed response, respectively [48]. These discoveries suggest that mitochondrial respiration and peroxisomal functions support LSEC-dependent immune responses, including antiviral immunity.

Besides the role of oxidative phosphorylation related to the scavenging properties and immune response of LSECs [20], there might be an additional reason for their reliance on oxidative phosphorylation related to the liver’s metabolic function. The concentrations of metabolic substrates (glucose, SCFAs and amino acids) in the portal vein fluctuate greatly depending on the fasting versus postprandial state of the organism [49, 50]. The arterial blood glucose concentration is typically around 5 mM and slightly less than 5 mM in a post-absorptive state. However, it can reach around 8 mM in peripheral blood and even around 10 mM in the portal vein in healthy individuals after a single meal [51]. It was shown in rats that glucose concentrations in the peripheral blood and portal vein were comparably low (< 5 mM) only under fasting conditions. However, significantly higher glucose levels were reached in the portal vein after a meal [52]. Similarly, higher glucose levels were observed in the portal vein than in the systemic circulation in newborn lambs fed mother’s milk [53]. The liver helps to regulate systemic fluxes in metabolic substrates [49].

Glycogen and FAs are used under fasting conditions, but hepatic glycogen synthesis and de novo lipogenesis are promoted under feeding conditions. Therefore, glucose storage and release are fundamental functions of the liver, minimising the changes in systemic glucose levels under fasting versus postprandial conditions [54]. Consequently, the liver has evolved to function under fluctuating glucose conditions. Furthermore, fluctuations in blood glucose concentrations in the rat thoracic aorta were observed to enhance monocyte adhesion to the aortic endothelium [55] and to increase oxidative stress, the inflammatory response and EC apoptosis [56]. Glycemic variability and hyperglycemia generally promote the development of endothelial dysfunction and cardiovascular diseases [57–60]. Consequently, the potential dependence of LSECs mainly on glycolysis for energy metabolism in vivo might not be optimal under fluctuating glucose levels. Therefore, oxidative phosphorylation appears to be a better strategy than glycolysis for ATP production in LSECs to maintain their viral endocytosis and immune response functions in the unique metabolic microenvironment of the liver. If so, it is tempting to speculate that preserving these functions could explain why prolonged exposure to an HFD in vivo did not induce permanent mitochondrial dysfunction in isolated LSECs; although, it did cause other phenotypic changes related to liver steatosis and endothelial inflammation [21]. Similarly, LSECs’ energy metabolism and endocytic capacity were not altered in the progression of heart failure (Tgαq*44 mice) despite their dysfunction involving defenestration and altered eicosanoid and proteomic profiles [61], as if their reliance on oxidative phosphorylation was the last function to fail. What mechanisms cause impaired oxidative phosphorylation in LSEC dysfunction and how they contribute to liver or systemic diseases remains to be determined.

### Metabolic plasticity of LSECs

The liver is supplied with various metabolic substrates through the portal vein, such as simple sugars (glucose and fructose), amino acids and SCFAs [49]. In isolated LSECs in vitro, we did not observe any substrate preferences even after applying a combination of different metabolic enzyme inhibitors (Additional file 1: Fig. S6). These results demonstrate the high metabolic plasticity of LSECs. However, these experiments were performed in a DMEM-based medium supplemented with glucose, glutamine and pyruvate, which also contained other constituents, including amino acids, making it impossible to draw firm conclusions from them. Therefore, we replaced the DMEM with KHB supplemented with glucose, which, based on these experiments, appeared necessary for LSEC mitochondrial respiration. Altogether, our results showed that glycolysis was not a preferential ATP-generating pathway in LSECs, but glucose was necessary for energy metabolism as a source of pyruvate. Notably, another source of pyruvate could be alanine, converted by glutamic-pyruvic transaminase [62]. However, even under a poor availability of exogenous metabolic substrates (only 2.5 mM glucose), the inhibitory effect of UK-5099 was abolished by l-carnitine (Fig. 3H, I), suggesting the high availability of endogenous metabolic substrates, most likely FAs. Interestingly, mitochondrial respiration appears more susceptible to metabolic inhibitors in blood platelets, which also show high metabolic plasticity [63], than in LSECs. Therefore, the metabolic plasticity of LSECs may be related to the liver environment or specific LSEC functions.

In summary, our study demonstrated that (1) LSECs have high metabolic plasticity, (2) glucose-derived pyruvate is an important metabolic fuel for LSEC oxidative phosphorylation and (3) LSECs contain high levels of endogenous FAs to support oxidative phosphorylation. Notably, these experiments clearly show the importance of controlling the assay medium composition for interpreting results related to the energy metabolism of cells.

### The importance of free FAs for LSEC energy metabolism

Among the FAs transported through the portal vein to the liver are SCFAs, MCFAs and LCFAs. The primary source of SCFAs in the liver portal vein is the gut microbiota [64, 65], while MCFAs and LCFAs are acquired predominantly from the diet [51, 66]. Both SCFAs and MCFAs are delivered as non-esterified FAs [51]. After the digestion of lipids in the small intestine, free LCFAs and monoacylglycerols are also re-esterified in enterocytes to form new triacylglycerol molecules, which are then packed into chylomicrons and sent primarily to lymphatic vessels. However, some LCFAs enter the portal vein directly [67]. Our results showed that LSECs preferred SCFAs and MCFAs over LCFAs for mitochondrial oxidation (Figs. 3, 5), which was not due to the lack of availability of LCFAs. Analysis of the acylcarnitine profile showed that PA was efficiently delivered to LSECs and processed (shortened) inside them (Fig. 5A). However, it was not directly oxidised in mitochondria, as shown by the lack of effects of ETO on oxygen consumption and the acylcarnitine profile (Fig. 5).

Here, the question arises: Why was the direct utilisation of LCFAs reduced in LSEC mitochondria in favour of MCFAs or SCFAs? Since FAO is associated with increased ROS production in mitochondria, FA-induced uncoupling and mitochondrial fragmentation are considered protective mechanisms [66, 68]. SCFAs and MCFAs contribute to energy dissipation not through their protonophoric abilities but the pseudo-uncoupling of ATP utilisation, relying on the activation and slow intramitochondrial hydrolysis of their activation products, acyl-AMP and acyl-CoA [66, 69]. Since LSECs are exposed to blood containing high concentrations of FAs and other metabolic substrates, a system is needed to protect against excessive ATP production in mitochondria, which would exceed the energetic demands of the cell and induce oxidative stress. The intracellular accumulation of PA in the form of triacylglycerides was shown to protect against FA-induced lipotoxicity [70]. In addition, PA was esterified and stored in LDs in our experimental model, as confirmed by Raman spectroscopy (Fig. 4). Furthermore, the low abundance of CPT1A (Additional file 1: Figs. S2G and S8) could be a way to reduce the direct transport of PA to mitochondria. Another possible mechanism inhibiting residual CPT1A activity could be malonyl-CoA [71, 72], but it remains to be tested in LSECs. Importantly, PA was still available as an energy source but most likely after shortening to MCFAs or SCFAs via β-oxidation in peroxisomes.

Peroxisomes generally oxidise very long-chain FAs, long-chain dicarboxylic FAs or polyunsaturated FAs, whereas mitochondria oxidise LCFAs, MCFAs and SCFAs [73]. However, β-oxidation in peroxisomes is important under mitochondrial β-oxidation deficiency or overload [74]. In our study, LSECs preferred to oxidise LCFAs in peroxisomes, most likely because of the FA overload. Our data indicate the importance of the metabolic interaction between peroxisomes and mitochondria. As Wanders et al. discussed, carnitine is unnecessary for FA uptake by peroxisomes [38]. However, it is required to transport shortened FAs generated in peroxisomes to mitochondria. In addition, FAs, which undergo shortening to acetyl-CoA, propionyl-CoA and MCFA-CoAs in peroxisomes, must be transported to mitochondria to be fully oxidised to carbon dioxide and water. Our data demonstrate relatively high levels of acetylcarnitine and propionylcarnitine (Additional file 1: Supplementary Table 1; only palmitoylcarnitine was higher than acetylcarnitine). Both can arise from FAO in peroxisomes: acetylcarnitine from even-chain FAs and propionylcarnitine from odd-chain FAs. However, propionylcarnitine can also originate from the catabolism of amino acids. Moreover, it can be an anaplerotic substrate for the TCA [75]. Importantly, it was shown in rat hearts that peroxisomal acetyl-CoA is a preferential source for synthesising malonyl-CoA, which regulates LCFA oxidation in mitochondria [76]. Based on our studies, we hypothesise that in LSECs, PA undergoes several cycles of peroxisomal β-oxidation to circumvent mitochondrial oxidation and the ensuing ROS production in the ETC.

The upregulation of CAT (Additional file 1: Fig. S2I), which allows an efficient and safe decomposition of hydrogen peroxide generated during the first peroxisomal β-oxidation reaction, suggests increased peroxisomal β-oxidation activity in LSECs. This strategy seems important for LSECs to reduce the intramitochondrial generation of ROS since the cells are overloaded with metabolic substrates because of the liver environment. Therefore, the oxidation of FAs, delivered to mitochondria as MCFAs or SCFAs instead of LCFAs, appears beneficial for LSECs. However, the functional cooperation between mitochondria and peroxisomes in LCFA processing [38] might also be important in the broader context of LSEC immune function. To definitively confirm the preprocessing of LCFAs in LSEC peroxisomes, further studies are needed in cells isolated from genetically modified animals with endothelium-specific peroxisomal β-oxidation inactivity, such as by disrupting peroxisome biogenesis or selectively inhibiting peroxisome functions.

### Different oxygen tensions in studies in primary LSECs

One limitation of this study that should be considered is that our experiments on LSECs were performed in vitro using imperfect models of isolated primary cells, with non-physiological mechanical stress, atmospheric oxygen levels, metabolic fuel concentrations and cellular environments (i.e. the lack of surrounding cells normally present in the liver). However, some of these limitations often raised in literature, related to atmospheric oxygen levels could be discussed. Oxygen tension in the liver is within the range of 30–90 mmHg [77], which is around 4–12%. Martinez et al. investigated the effects of incubating LSECs under 5% versus 20% oxygen tension with a glucose concentration (around 12 mM) corresponding to the postprandial state. They demonstrated that 5% oxygen tension improved the viability, structure and function of LSECs [77]. Furthermore, LSECs incubated under 20% oxygen tension produced more lactate but did not consume more glucose. Since the publication of these results, various research groups have used conditions of 5% [46, 78] or atmospheric [20, 79, 80] oxygen tension in their studies of LSEC biology.

Our results obtained at atmospheric oxygen tension agree with recently published works by Li et al., who showed enhanced glycolysis and reductions in processes related to oxidative phosphorylation in the metabolism of primary rat LSECs from 2 to 24 h in culture at the protein and mRNA levels [78] and by Dudek et al., who showed that mitochondrial respiration prevails over glycolysis in LSECs [20]. We recently demonstrated that energy metabolism was preserved in LSECs isolated from a murine model of chronic heart failure (Tgαq*44 mice) compared with control (FVB) mice when incubated overnight under 5% oxygen tension [61]. Notably, the rates and profiles of mitochondrial respiration and glycolysis in the work by Wojnar-Lason et al.  were comparable to our results in this study.

The increase in oxygen availability may not significantly alter LSEC energy metabolism. Most types of cells adjust the rates of mitochondrial respiration and glycolysis to cellular energy demands, not to the increased availability of metabolic substrates [81]. However, the opposite situation, where cells adjust the rate of metabolism to the increased availability of metabolic substrates, is also possible. For example, pancreatic β-cells increase oxygen metabolism in response to elevated extracellular glucose availability to promote glucose-stimulated insulin secretion [82]. Further in vivo studies are needed to exclude the influence of manipulation procedures and changes in environmental conditions accompanying the isolation of cells from their vascular bed on the energy metabolism of different types of ECs. Such studies should definitively determine what types of energy metabolism ECs prefer in different vascular beds.

In conclusion, despite these limitations, our experiments provided novel insights into the bioenergetic profile of primary murine LSECs that rely more on oxidative phosphorylation and less on glycolysis for ATP production. While glycolysis was not a primary ATP-generating pathway in LSECs and the cells showed high metabolic plasticity, glucose was an important metabolic substrate. Despite the high availability of LCFAs for oxidative phosphorylation in LSEC mitochondria, the preferentially used energy substrates appeared to be SCFAs and MCFAs, which could have been preprocessed from LCFAs in peroxisomes before entering mitochondria. The transfer of LCFA oxidation from mitochondria to peroxisomes enables the relocation of ROS-generating reactions from vulnerable mitochondria to CAT-protected peroxisomes. Together with the extensive redox homeostasis machinery, this strategy could protect LSECs against overload with metabolic fuels in the liver environment. A better understanding of the mechanisms of LSEC resistance to the overload with metabolic substrates would be helpful in developing new strategies to protect different types of ECs from developing endothelial dysfunction in cardiometabolic diseases. Finally, the reliance of LSECs on oxidative phosphorylation with high metabolic plasticity distinguishes them from the various ECs present in other vascular beds. The functional relevance of this unusual bioenergetic profile of LSECs to health and disease remains to be determined.

### Supplementary Information

**Additional file 1: Figure S1.** Marker proteins in LSECs. Heat maps of the relative abundance (emPAI%) of marker proteins confirming LSEC purity. STAB2, Stabilin-2; OIT3, Oncoprotein-induced transcript 3; CLEC4G, C-type lectin domain family 4 member G; CLEC1B, C-type lectin domain family 1 member B; F8, Coagulation factor VIII; LYVE1, Lymphatic vessel endothelial hyaluronic acid receptor 1; FCGR2, Low affinity immunoglobulin gamma Fc region receptor II; EHD3, EH domain-containing protein 3; MRC1, Macrophage mannose receptor 1 (CD206); STAB1, Stabilin-1; PTPRB, Receptor-type tyrosine-protein phosphatase beta; AKAP12, A-kinase anchor protein 12; DAB2, Disabled homolog 2; ACP5, Tartrate-resistant acid phosphatase type 5; CD36, Platelet glycoprotein 4; FLT4, Vascular endothelial growth factor receptor 3; GPR182, G-protein coupled receptor 182; CDH5, Cadherin-5; MYCT1, Myc target protein 1; ADGRF5, Adhesion G protein-coupled receptor F5; ADGRL4, Adhesion G protein-coupled receptor L4; ENG, Endoglin; IGFBP7, Insulin-like growth factor-binding protein 7; PLP3, Phospholipid phosphatase 3. Data come from eight replicates. **Figure S2.** Energy metabolism enzymes in LSECs; proteomic analysis. (A-G; I-L) Heat maps of the relative abundance (emPAI%) of proteins identified in LSECs related to the glycolysis (A), TCA cycle and complex II of the ETC (B), complex I of the ETC (C), complex III of the ETC (D), complex IV of the ETC (E), complex V of the ETC (F), FA oxidation in mitochondria (G), peroxisomes and FA oxidation in peroxisomes (I), pyruvate metabolism (J), glutamine metabolism (K), and malate-aspartate shuttle (L). Data come from eight replicates. (H) Relative abundance (emPAI%) of 3-ketoacyl-CoA thiolase in LSECs; this component of the β-oxidation pathway shown in G is presented on a separate graph due to its abundance, which was far superior to the rest of the proteins identified in this pathway. Data come from eight replicates. **Figure S3.** Gene Ontology (GO)-enrichment proteomics analysis in LSECs. Gene enrichment analysis of proteins in LSECs. Functional enrichment analysis showing 20 the most significant categories of cellular components (A), biological processes (B) and molecular functions (C). Hierarchical clustering was performed with ShinyGO software 0.77. The charts provide information about GO fold enrichment (y-axis; pathways in order of the enrichment of FDR (false discovery rate)), significance (x-axis; FDR in log10), and number of proteins in each pathway. The color charts show the fold enrichment for each pathway. The size of dots corresponds to the number of genes assigned to each pathway. Data come from eight replicates for each type of cells. **Figure S4.** Assessment of oxidative phosphorylation and glycolysis in HAECs. A Heat map of the relative abundance (emPAI%) of selected proteins identified in HAECs related to the TCA cycle, ETC, FA oxidation in mitochondria (FAO), malate–aspartate shuttle (MAS), glutamine metabolism (G), peroxisomes and FA oxidation in peroxisomes (PX), and glycolysis. Data come from eight replicates. B Representative image of HAEC mitochondria. Mitochondria (green) were labelled with MitoTracker Green FM, and nuclei (blue) were labelled with Hoechst 33342. C Changes in ATP concentration in HAECs treated for 1 h with DMSO (as a vehicle), oligomycin (O; 1 µg/mL), or IAA (20 µM). Data are shown as means ± SEMs from three independent experiments (with three replicates per experiment). Significance was tested using one-way ANOVA (A and B); *, *p* < 0.05. D The OCR (grey) and ECAR (blue) measured using the MST in HAECs; the mitochondrial function parameters were calculated from the kinetic data. Data are shown as means ± SEMs from three independent experiments (with 5–6 replicates per experiment). E The ECAR (blue) and OCR (grey) measured using the GST in HAECs; the glycolytic function parameters were calculated from the kinetic data. Data are shown as means ± SEMs from three independent experiments (with 5–6 replicates per experiment). **Figure S5.** Assessment of mitochondrial and glycolytic function in LSECs 2 h after cell isolation. A The OCR (grey) and ECAR (green) measured using the MST in LSECs 2 h after cell isolation. Data are shown as means ± SEMs from four biological replicates (with 5-6 technical replicates per biological replicate). B The ECAR (green) and OCR (grey) measured using the GST in LSECs 2 h after cell isolation. Data are shown as means ± SEMs from four biological replicates (with 5-6 technical replicates per biological replicate). **Figure S6.** Metabolic plasticity of LSECs. The OCR measured using the MFFT in LSECs in unbuffered DMEM supplemented with 5.5 mM glucose, 2 mM glutamine, and 1 mM pyruvate using the following reagents: UK-5099 (2 µM), BPTES (3 µM), and ETO (4 µM). Data are shown as means ± SEMs from four independent experiments (with 3–6 replicates per experiment). **Figure S7.** Effects of CPT1 inhibitors. (A and B) The OCR measured in LSECs in KHB supplemented with glucose (2.5 mM), untreated or treated with PER (5 µM; A) or OXF (1 mM; B), followed by DMSO or UK-5099 (10 µM) and CB-839 (5 µM), then FCCP (2 µM) and R/A (0.5/ 0.5 µM). Changes in basal respiration (90 min after PER or OXF addition) and maximal OCR were calculated from the kinetic data. Data are shown as means ± SEMs from three independent experiments (with 3-4 replicates per experiment). (C and D) The OCR measured in LSECs in KHB supplemented with glucose (2.5 mM), L-carnitine (50 µM) and PA-BSA (20 µM/3.3 µM), untreated or treated with PER (5 µM; A) or OXF (1 mM; B), followed by DMSO or UK-5099 (10 µM) and CB-839 (5 µM), then FCCP (5 µM) and R/A (1/1 µM). Changes in basal respiration (90 min after addition of PER or OXF) and maximal OCR were calculated from the kinetic data. Data are shown as means ± SEMs from three independent experiments (with 3-4 replicates per experiment). (E and F) The OCR measured in LSECs in KHB supplemented with glucose (2.5 mM), L-carnitine (50 µM) and BSA (3.3 µM), untreated or treated with PER (5 µM; A) or OXF (1 mM; B), followed by DMSO or UK-5099 (10 µM) and CB-839 (5 µM), then FCCP (5 µM) and R/A (1/1 µM). Changes in basal respiration (90 min after addition of PER or OXF) and maximal OCR were calculated from the kinetic data. Data are shown as means ± SEMs from three independent experiments (with 3-4 replicates per experiment). **Figure S8.** Carnitine palmitoyltranserase 1A. Relative CPT1A levels were quantified by Western blot analysis in protein lysates prepared from breast cancer MCF-7 cells, HAECs and LSECs. The relative levels of CPT1A were quantified by normalizing to the loading control. The protein concentration in each cell lysate was measured by Pierce BCA Protein Assay Kit (Thermo Fisher Scientific). Samples containing 10 μg of total proteins were loaded into 7.5% SDS-PAGE gel, separated electrophoretically, and transferred onto PVDF membranes (Bio-Rad). Next, the membranes were blocked with 5% dry milk, and incubated overnight with the primary antibody (1:000) directed against the CPT1A (ab234111, Abcam) and with mouse IgG kappa binding protein (mIgGκ BP) conjugated to horseradish peroxidase (sc-516102, Santa Cruz Biotechnology), 1:5000 for 1 hour. Immunoreactive band detection was achieved by chemiluminescence (ChemiDocMP; Bio-Rad). Densitometric band analysis was performed using the ImageJ software. The total protein loaded onto the particular lane after transfer was used as the loading control using stain-free technology (Bio-Rad). **Supplementary Table 1.** Energy metabolism enzymes in LSECs; proteomic analysis. **Supplementary Table 2.** Energy metabolism enzymes in HAECs; proteomic analysis. **Supplementary Table 3.** Acylcarnitines levels in LSECs.

**Additional file 2:** Key resources table.

## Data Availability

The raw data for all experiments will be made available upon request.

## Abbreviations

ACOX1: Acyl-CoA oxidase 1
BA: Butyric acid
BPTES: Glutaminase 1 inhibitor (bis-2-[5-phenylacetamido-1,3,4-thiadiazol-2-yl]ethyl sulfide)
BSA: Bovine serum albumin
CAT: Catalase
CB-839: Glutaminase 1 inhibitor (N-[5-[4-[6-[[2-[3-(trifluoromethoxy)phenyl]acetyl]amino]-3-pyridazinyl]butyl]-1,3,4-thiadiazol-2-yl]-2-pyridineacetamide)
CoA: Coenzyme A
CPT1A: Carnitine palmitoyltransferase 1A
DMEM: Dulbecco’s Modified Eagle’s Medium
DMSO: Dimethyl sulfoxide
d-PA: Deuterium labelled palmitic acid
ECAR: Extracellular acidification rate
ECs: Endothelial cells
ETC: Electron transport chain
ETF: Electron transfer flavoprotein
ETO: Etomoxir
FAs: Fatty acids
FAO: Fatty acid oxidation
FCCP: Mitochondrial uncoupler (carbonyl cyanide 4-[trifluoromethoxy]phenylhydrazone)
GAPDH: Glyceraldehyde-3-phosphate dehydrogenase
GLS1: Glutaminase 1
GLUD1: Glutamate dehydrogenase 1
GOT2: Glutamic-oxaloacetic transaminase 2
GST: Glycolysis stress test
HAECs: Human aortic endothelial cells
HFD: High-fat diet
IAA: Iodoacetic acid
KHB: Krebs-HEPES buffer
LCFAs: Long-chain fatty acids
LC–MS/MS: Liquid chromatography-tandem mass spectrometry
LDs: Lipid droplets
LSECs: Liver sinusoidal endothelial cells
MAS: Malate–aspartate shuttle
MCFAs: Medium-chain fatty acids
MFFT: Mitochondrial fuel flexibility test
MPC: Mitochondrial pyruvate carrier
MST: Mitochondrial stress test
OA: Octanoic acid
OCR: Oxygen consumption rate
PA: Palmitic acid
PA-BSA: Palmitic acid conjugated with bovine serum albumin
PER: Perhexiline
R/A: Rotenone with antimycin A
ROS: Reactive oxygen species
SCFAs: Short-chain fatty acids
TCA: Tricarboxylic acid cycle
UK-5099: Mitochondrial pyruvate carrier inhibitor (2-cyano-3-[1-phenyl-1H-indol-3-yl]-2-propenoic acid)
UK/CB: UK-5099 and CB-839
