# Supplementary material for: Liver sinusoidal endothelial cells rely on oxidative phosphorylation but avoid processing long-chain fatty acids in their mitochondria
Source: Cell Mol Biol Lett. 2024 May 9;29:67. doi: 10.1186/s11658-024-00584-8 (PMC11084093; doi:10.1186/s11658-024-00584-8)
Supplement: Supplementary file 1 — Additional file 1: Figure S1. Marker proteins in LSECs. Heat maps of the relative abundance (emPAI%) of marker proteins confirming LSEC purity. STAB2, Stabilin-2; OIT3, Oncoprotein-induced transcript 3; CLEC4G, C-type lectin domain family 4 member G; CLEC1B, C-type lectin domain family 1 member B; F8, Coagulation factor VIII; LYVE1, Lymphatic vessel endothelial hyaluronic acid receptor 1; FCGR2, Low affinity immunoglobulin gamma Fc region receptor II; EHD3, EH domain-containing protein 3; MRC1, Macrophage mannose receptor 1 (CD206); STAB1, Stabilin-1; PTPRB, Receptor-type tyrosine-protein phosphatase beta; AKAP12, A-kinase anchor protein 12; DAB2, Disabled homolog 2; ACP5, Tartrate-resistant acid phosphatase type 5; CD36, Platelet glycoprotein 4; FLT4, Vascular endothelial growth factor receptor 3; GPR182, G-protein coupled receptor 182; CDH5, Cadherin-5; MYCT1, Myc target protein 1; ADGRF5, Adhesion G protein-coupled receptor F5; ADGRL4, Adhesion G protein-coupled receptor L4; ENG, Endoglin; IGFBP7, Insulin-like growth factor-binding protein 7; PLP3, Phospholipid phosphatase 3. Data come from eight replicates. Figure S2. Energy metabolism enzymes in LSECs; proteomic analysis. (A-G; I-L) Heat maps of the relative abundance (emPAI%) of proteins identified in LSECs related to the glycolysis (A), TCA cycle and complex II of the ETC (B), complex I of the ETC (C), complex III of the ETC (D), complex IV of the ETC (E), complex V of the ETC (F), FA oxidation in mitochondria (G), peroxisomes and FA oxidation in peroxisomes (I), pyruvate metabolism (J), glutamine metabolism (K), and malate-aspartate shuttle (L). Data come from eight replicates. (H) Relative abundance (emPAI%) of 3-ketoacyl-CoA thiolase in LSECs; this component of the β-oxidation pathway shown in G is presented on a separate graph due to its abundance, which was far superior to the rest of the proteins identified in this pathway. Data come from eight replicates. Figure S3. Gene Ontology (GO)-enrichment proteo [file 11658_2024_584_MOESM1_ESM.pdf]

Supplementary Material

Liver sinusoidal endothelial cells rely on oxidative phosphorylation but avoid processing long-chain fatty acids in their mitochondria

Patrycja Kaczara[a*], Izabela Czyzynska-Cichon[a], Edyta Kus[a], Anna Kurpinska[a], Mariola Olkowicz[a], Kamila Wojnar-Lason[a,b], Marta Z. Pacia[a], Olena Lytvynenko[a], Myriam Baes[c], and Stefan Chlopicki[a,b]

[a]Jagiellonian University, Jagiellonian Centre for Experimental Therapeutics (JCET), Krakow 30-348, Poland

[b]Jagiellonian University Medical College, Department of Pharmacology, Krakow 31-531, Poland

[c]KU Leuven, Department of Pharmaceutical and Pharmacological Sciences, Laboratory of Cell Metabolism, 3000 Leuven, Belgium

*Corresponding author:

Patrycja Kaczara, Jagiellonian University, Jagiellonian Centre for Experimental Therapeutics, Bobrzynskiego 14, 30-348 Krakow, Poland; tel: +48 12 6645464; fax: +48 12 2974615; email: patrycja.kaczara@jcet.eu; ORCID ID: 0000-0003-0106-4172

Figure S1

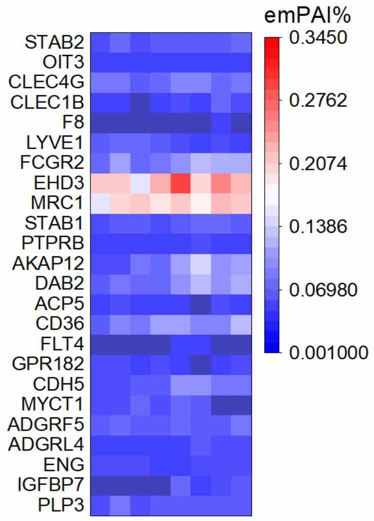

Figure S1. Marker proteins in LSECs

Heat maps of the relative abundance (emPAI%) of marker proteins confirming LSEC purity. STAB2 – Stabilin-2, OIT3 – Oncoprotein-induced transcript 3, CLEC4G – C-type lectin domain family 4 member G, CLEC1B – C-type lectin domain family 1 member B, F8 – Coagulation factor VIII, LYVE1 – Lymphatic vessel endothelial hyaluronic acid receptor 1, FCGR2 – Low affinity immunoglobulin gamma Fc region receptor II, EHD3 – EH domain-containing protein 3, MRC1 – Macrophage mannose receptor 1 (CD206), STAB1 – Stabilin-1, PTPRB – Receptor-type tyrosine-protein phosphatase beta, AKAP12 – A-kinase anchor protein 12, DAB2 – Disabled homolog 2, ACP5 – Tartrate-resistant acid phosphatase type 5, CD36 – Platelet glycoprotein 4, FLT4 – Vascular endothelial growth factor receptor 3, GPR182 – G-protein coupled receptor 182, CDH5 – Cadherin-5, MYCT1 – Myc target protein 1, ADGRF5 – Adhesion G protein-coupled receptor F5, ADGRL4 – Adhesion G protein-coupled receptor L4, ENG – Endoglin, IGFBP7 – Insulin-like growth factor-binding protein 7, PLP3 – Phospholipid phosphatase 3. Data come from eight replicates.

Figure S2

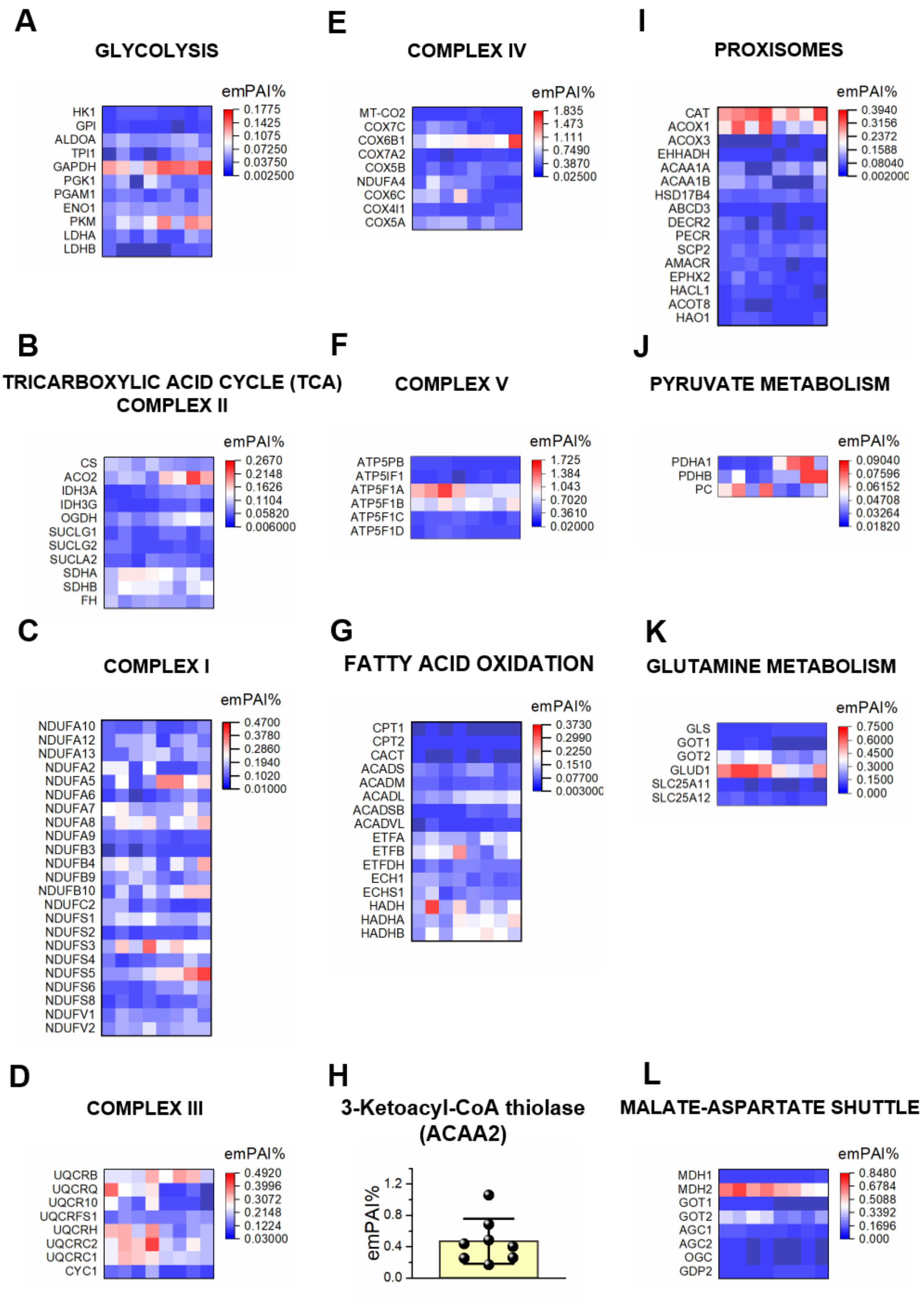

Figure S2. Energy metabolism enzymes in LSECs; proteomic analysis.

(A-G; I-L) Heat maps of the relative abundance (emPAI%) of proteins identified in LSECs related to the glycolysis (A), TCA cycle and complex II of the ETC (B), complex I of the ETC (C), complex III of the ETC (D), complex IV of the ETC (E), complex V of the ETC (F), FA oxidation in mitochondria (G),

peroxisomes and FA oxidation in peroxisomes (I), pyruvate metabolism (J), glutamine metabolism (K), and malate-aspartate shuttle (L). Data come from eight replicates.

(H) Relative abundance (emPAI%) of 3-ketoacyl-CoA thiolase in LSECs; this component of the β-oxidation pathway shown in G is presented on a separate graph due to its abundance, which was far superior to the rest of the proteins identified in this pathway. Data come from eight replicates.

Figure S3

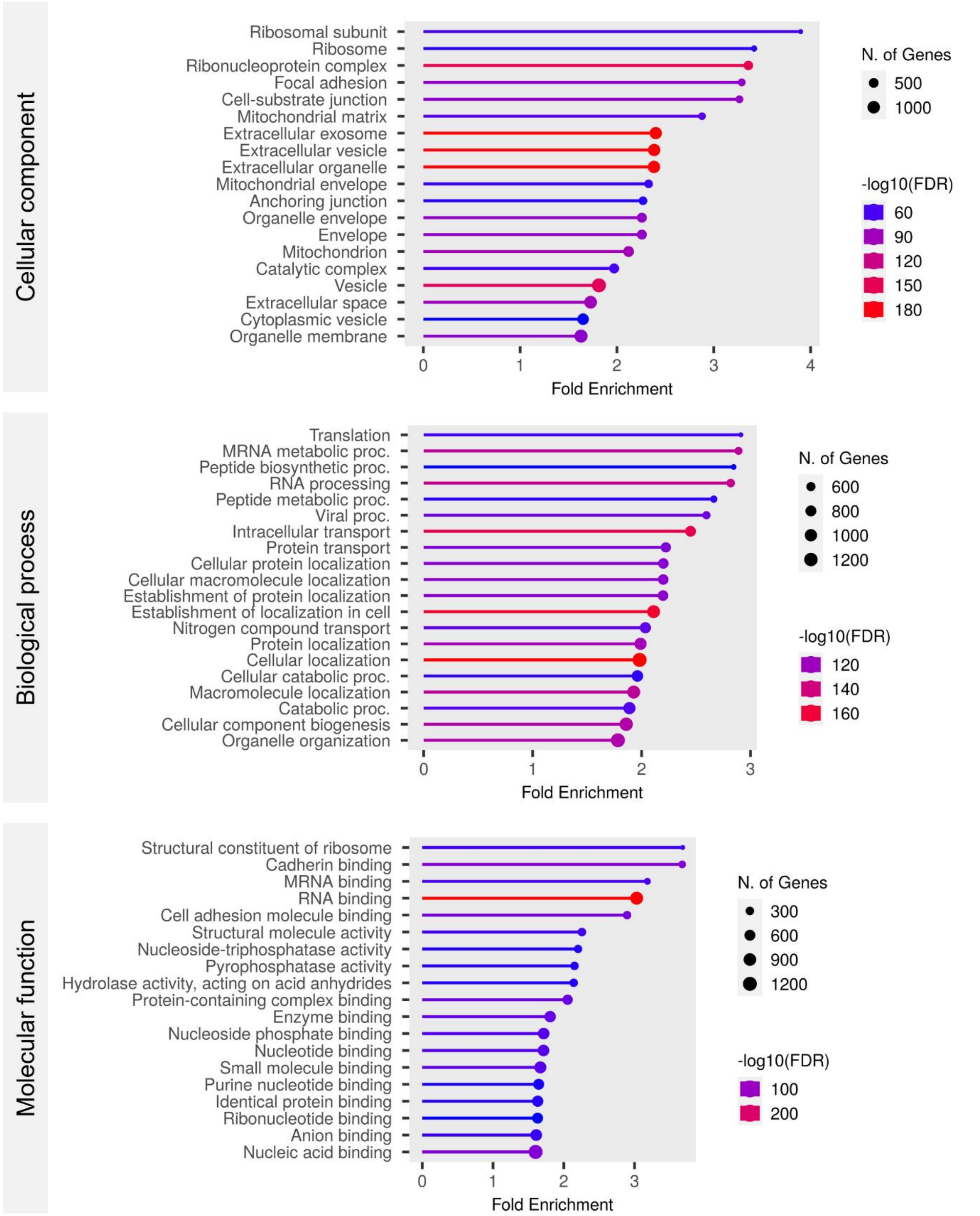

Figure S3. Gene Ontology (GO)-enrichment proteomics analysis in LSECs

Gene enrichment analysis of proteins in LSECs. Functional enrichment analysis showing 20 the most significant categories of cellular components (A), biological processes (B) and molecular functions

(C). Hierarchical clustering was performed with ShinyGO software 0.77. The charts provide information about GO fold enrichment (y-axis; pathways in order of the enrichment of FDR (false discovery rate)), significance (x-axis; FDR in log10), and number of proteins in each pathway. The color charts show the fold enrichment for each pathway. The size of dots corresponds to the number of genes assigned to each pathway. Data come from eight replicates for each type of cells.

Figure S4

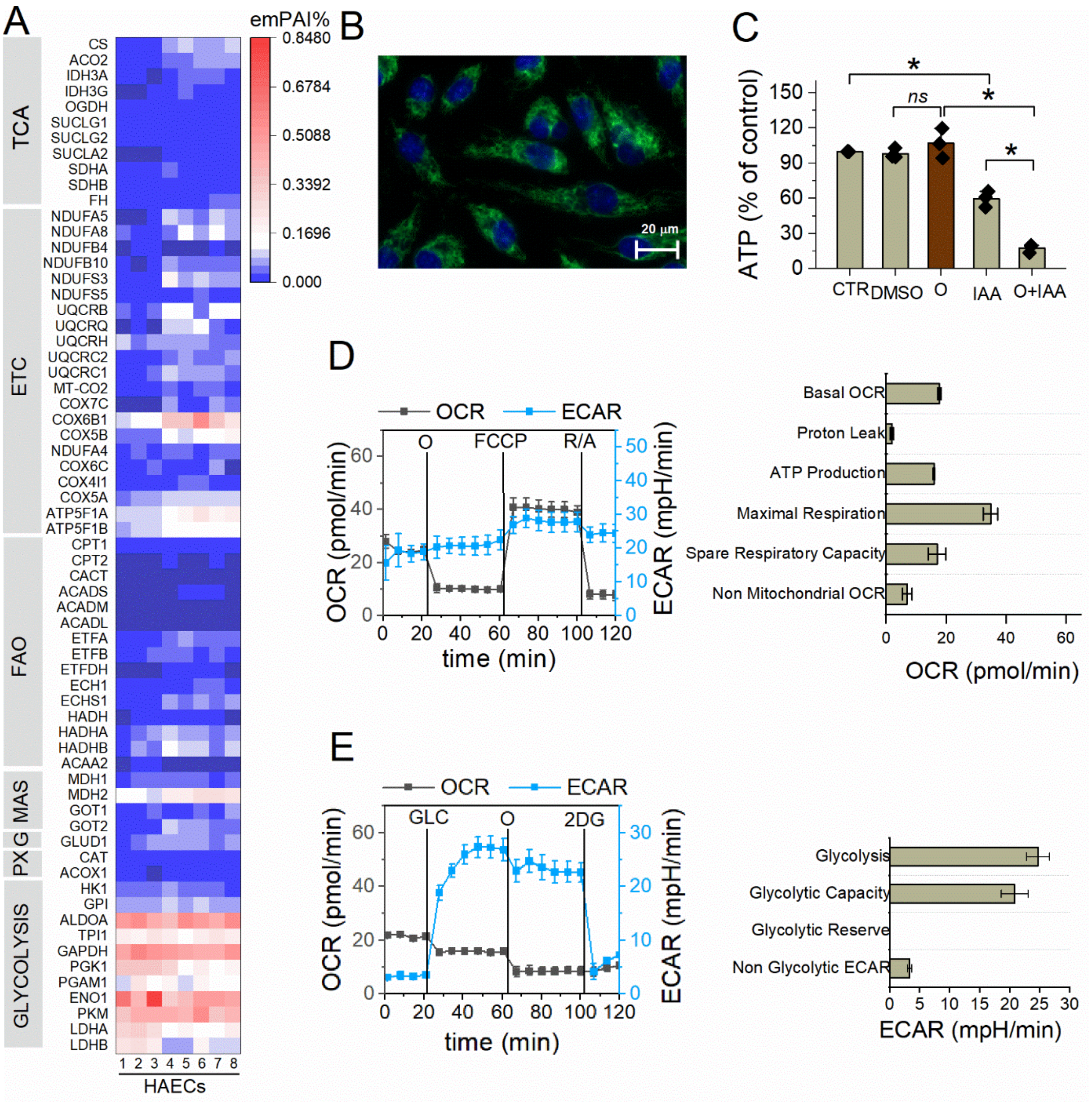

Figure S4. Assessment of oxidative phosphorylation and glycolysis in HAECs.

(A) Heat map of the relative abundance (emPAI%) of selected proteins identified in HAECs related to the TCA cycle, ETC, FA oxidation in mitochondria (FAO), malate-aspartate shuttle (MAS), glutamine metabolism (G), peroxisomes and FA oxidation in peroxisomes (PX), and glycolysis. Data come from eight replicates.

(B) Representative image of HAEC mitochondria. Mitochondria (green) were labelled with MitoTracker Green FM, and nuclei (blue) were labelled with Hoechst 33342.

(C) Changes in ATP concentration in HAECs treated for 1 h with DMSO (as a vehicle), oligomycin (O; 1 μg/mL), or IAA (20 μM). Data are shown as means ± SEMs from three independent experiments (with three replicates per experiment). Significance was tested using one-way ANOVA (A and B); *, $p < 0.05$.

(D) The OCR (grey) and ECAR (blue) measured using the MST in HAECs; the mitochondrial function parameters were calculated from the kinetic data. Data are shown as means ± SEMs from three independent experiments (with 5–6 replicates per experiment).

(E) The ECAR (blue) and OCR (grey) measured using the GST in HAECs; the glycolytic function parameters were calculated from the kinetic data. Data are shown as means ± SEMs from three independent experiments (with 5–6 replicates per experiment).

Figure S5

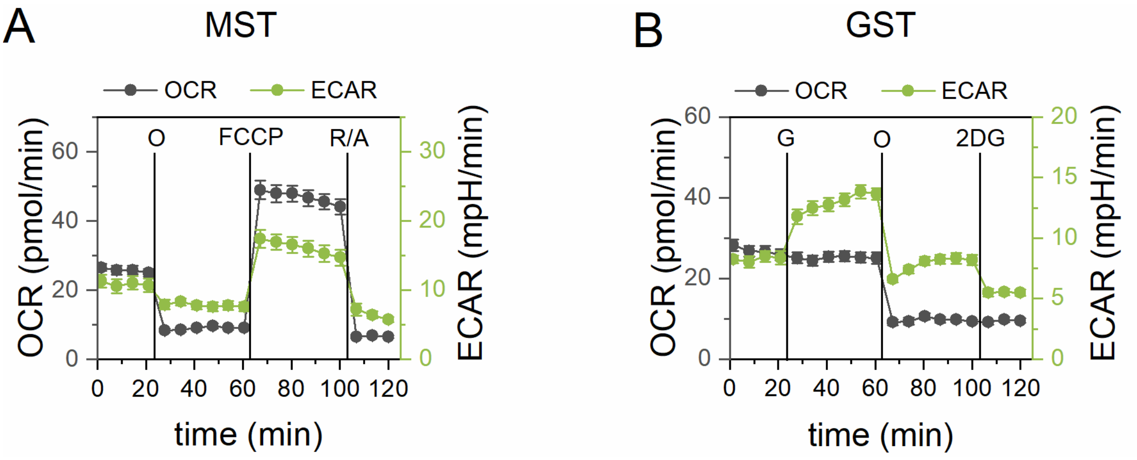

Figure S5. Assessment of mitochondrial and glycolytic function in LSECs 2 h after cell isolation

(A) The OCR (grey) and ECAR (green) measured using the MST in LSECs 2 h after cell isolation. Data are shown as means ± SEMs from four biological replicates (with 5-6 technical replicates per biological replicate).

(B) The ECAR (green) and OCR (grey) measured using the GST in LSECs 2 h after cell isolation. Data are shown as means ± SEMs from four biological replicates (with 5-6 technical replicates per biological replicate).

Figure S6

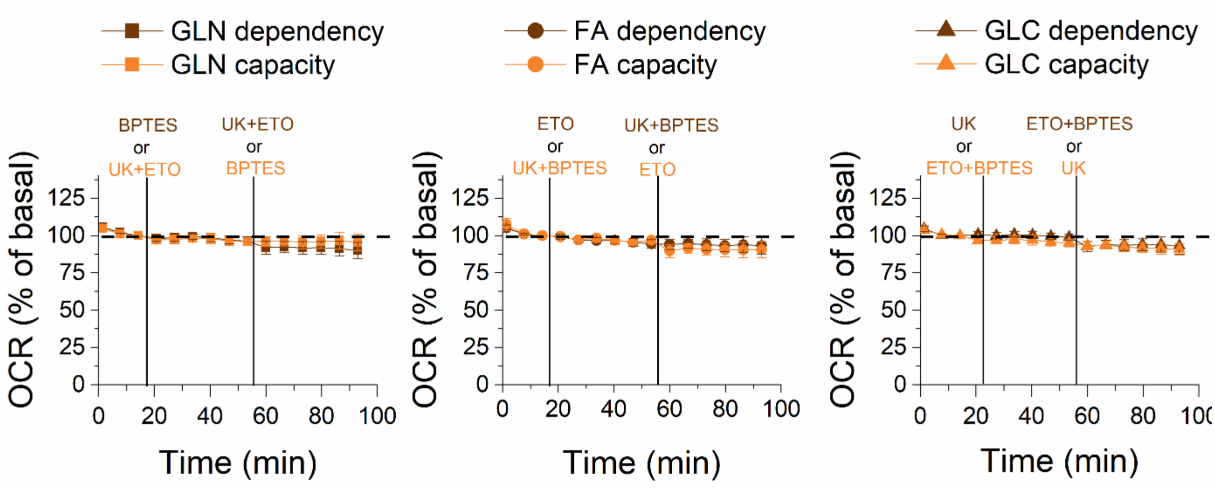

Figure S6. Metabolic plasticity of LSECs.

The OCR measured using the MFFT in LSECs in unbuffered DMEM supplemented with 5.5 mM glucose, 2 mM glutamine, and 1 mM pyruvate using the following reagents: UK-5099 (2 µM), BPTES (3 µM), and ETO (4 µM). Data are shown as means ± SEMs from four independent experiments (with 3–6 replicates per experiment).

Figure S7

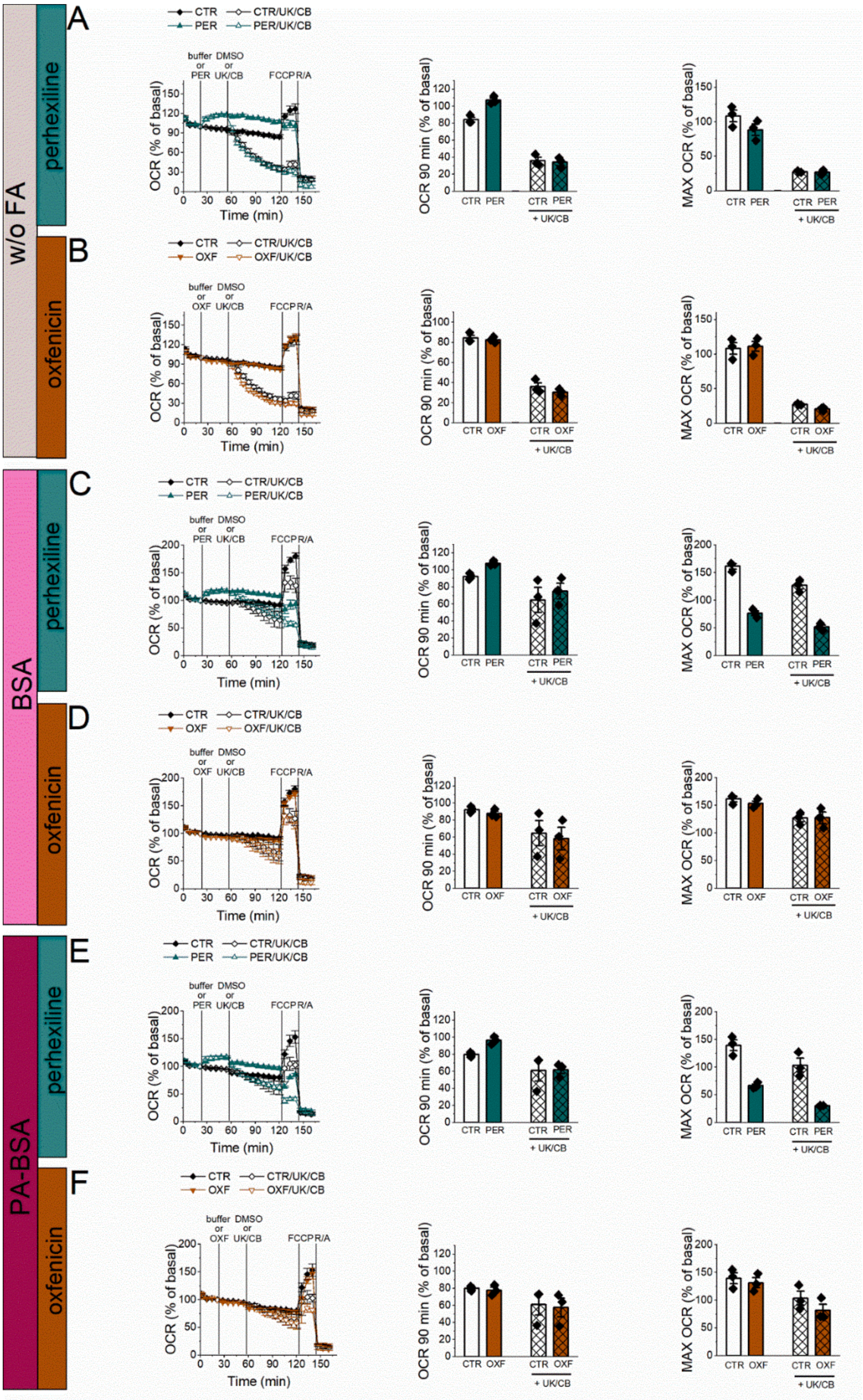

Figure S7. Effects of CPT1 inhibitors

(A and B) The OCR measured in LSECs in KHB supplemented with glucose (2.5 mM), untreated or treated with PER (5 µM; A) or OXF (1 mM; B), followed by DMSO or UK-5099 (10 µM) and CB-839 (5 µM), then FCCP (2 µM) and R/A (0.5/ 0.5 µM). Changes in basal respiration (90 min after PER

or OXF addition) and maximal OCR were calculated from the kinetic data. Data are shown as means ± SEMs from three independent experiments (with 3-4 replicates per experiment).

(C and D) The OCR measured in LSECs in KHB supplemented with glucose (2.5 mM), L-carnitine (50 µM) and PA-BSA (20 µM/3.3 µM), untreated or treated with PER (5 µM; A) or OXF (1 mM; B), followed by DMSO or UK-5099 (10 µM) and CB-839 (5 µM), then FCCP (5 µM) and R/A (1/1 µM). Changes in basal respiration (90 min after addition of PER or OXF) and maximal OCR were calculated from the kinetic data. Data are shown as means ± SEMs from three independent experiments (with 3-4 replicates per experiment).

(E and F) The OCR measured in LSECs in KHB supplemented with glucose (2.5 mM), L-carnitine (50 µM) and BSA (3.3 µM), untreated or treated with PER (5 µM; A) or OXF (1 mM; B), followed by DMSO or UK-5099 (10 µM) and CB-839 (5 µM), then FCCP (5 µM) and R/A (1/1 µM). Changes in basal respiration (90 min after addition of PER or OXF) and maximal OCR were calculated from the kinetic data. Data are shown as means ± SEMs from three independent experiments (with 3-4 replicates per experiment).

Figure S8

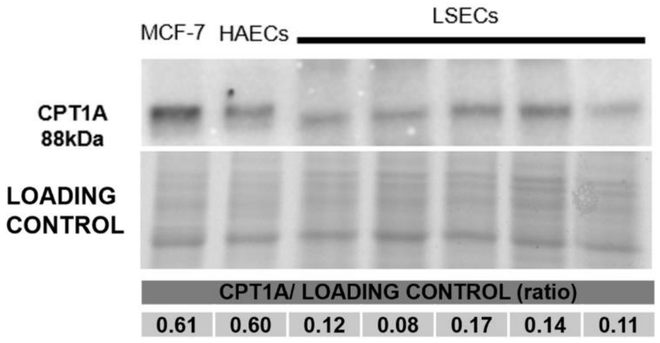

Figure S8. Carnitine palmitoyltranserase 1A

Relative CPT1A levels were quantified by Western blot analysis in protein lysates prepared from breast cancer MCF-7 cells, HAECs and LSECs . The relative levels of CPT1A were quantified by normalizing to the loading control. The protein concentration in each cell lysate was measured by Pierce BCA Protein Assay Kit (Thermo Fisher Scientific). Samples containing 10 µg of total proteins were loaded into 7.5% SDS-PAGE gel, separated electrophoretically, and transferred onto PVDF membranes (Bio-Rad). Next, the membranes were blocked with 5% dry milk, and incubated overnight with the primary antibody (1:000) directed against the CPT1A (ab234111, Abcam) and with mouse IgG kappa binding protein (m-IgGκ BP) conjugated to horseradish peroxidase (sc-516102, Santa Cruz Biotechnology), 1:5000 for 1 hour. Immunoreactive band detection was achieved by chemiluminescence (ChemiDocMP; Bio-Rad). Densitometric band analysis was performed using the ImageJ software. The total protein loaded onto the particular lane after transfer was used as the loading control using stain-free technology (Bio-Rad).

Table S1. Energy metabolism enzymes in LSECs; proteomic analysis

The relative abundance (emPAI%) of proteins identified in LSECs related to the energy metabolism pathways, presented also in reference (ratio) to GAPDH abundance. Data are shown as means ± SDs from eight replicates.

LSECs				emPAI%		ratio (GAPDH)	
Process	Protein	N Analysis	N Missing	Mean	SD	Mean	SD
GLYCOLYSIS	LDHB	4	4	0.02046	0.00345	0.14	0.034
	LDHA	8	0	0.02666	0.01604	0.20	0.115
	PKM	8	0	0.08998	0.04269	0.67	0.256
	ENO1	8	0	0.0433	0.0057	0.34	0.112
	PGAM1	8	0	0.02668	0.01213	0.21	0.118
	PGK1	7	1	0.04236	0.01977	0.31	0.178
	GAPDH	8	0	0.13634	0.03365	1.00	0
	TPI1	6	2	0.03274	0.01781	0.24	0.109
	ALDOC	0	8	--	--	--	--
	ALDOA	8	0	0.0432	0.0107	0.34	0.109
	PFKP	0	8	--	--	--	--
	PFKL	0	8	--	--	--	--
	PFKM	0	8	--	--	--	--
	GPI	7	1	0.00644	0.00261	0.05	0.029
	HK1	8	0	0.0121	0.00551	0.10	0.065
TCA	FH	8	0	0.072119	0.012761	0.57	0.236
	SDHB	8	0	0.114298	0.026322	0.92	0.438
	SDHA	8	0	0.122338	0.032383	1.02	0.596
	SUCLA2	8	0	0.033069	0.01582	0.24	0.102
	SUCLG2	8	0	0.028261	0.010432	0.22	0.093
	SUCLG1	8	0	0.031741	0.014507	0.24	0.108
	OGDH	8	0	0.082169	0.031756	0.60	0.175
	IDH3G	8	0	0.018679	0.011801	0.14	0.075
	IDH3B	0	8	--	--	--	--
	IDH3A	8	0	0.040705	0.02272	0.29	0.128
	ACO2	8	0	0.136109	0.074648	0.99	0.458
	CS	8	0	0.075166	0.019085	0.60	0.244
COMPLEX I	NDUFV2	8	0	0.1403	0.03328	1.11	0.437
	NDUFV1	8	0	0.105584	0.030068	0.85	0.406
	NDUFS8	8	0	0.05793	0.027083	0.43	0.160
	NDUFS6	8	0	0.089331	0.04549	0.63	0.238
	NDUFS5	8	0	0.243233	0.134105	1.75	0.713
	NDUFS4	8	0	0.082783	0.045116	0.60	0.278
	NDUFS3	8	0	0.25377	0.089171	1.96	0.793
	NDUFS2	8	0	0.042033	0.02211	0.30	0.145
	NDUFS1	8	0	0.166913	0.038683	1.36	0.674
	NDUFC2	8	0	0.066374	0.049644	0.52	0.408
	NDUFB10	8	0	0.181685	0.097433	1.31	0.573
	NDUFB9	8	0	0.114963	0.03973	0.99	0.723
	NDUFB4	8	0	0.194039	0.084017	1.52	0.712
	NDUFB3	6	0	0.042878	0.01911	0.31	0.186
	NDUFA9	8	0	0.05815	0.017126	0.48	0.275
	NDUFA8	8	0	0.219859	0.074228	1.73	0.765
	NDUFA7	8	0	0.175983	0.069186	1.39	0.663
	NDUFA6	7	1	0.064043	0.038778	0.44	0.277
	NDUFA5	8	0	0.215444	0.140813	1.47	0.822
	NDUFA2	8	0	0.111013	0.093676	0.87	0.755
	NDUFA13	8	0	0.124989	0.046673	1.00	0.547
	NDUFA12	8	0	0.112546	0.051001	0.94	0.616
	NDUFA10	8	0	0.064406	0.027237	0.49	0.199
COMPLEX III	CYC1	8	0	0.070506	0.041617	0.57	0.362
	UQCRC1	8	0	0.24746	0.070485	2.09	1.315
	UQCRC2	8	0	0.277639	0.114582	2.32	1.443
	UQCRH	8	0	0.229509	0.117933	1.86	1.093
	UQCRFS1	8	0	0.111399	0.022061	0.89	0.374
	UQCR10	7	1	0.124349	0.100637	1.00	0.769
	UQCRQ	7	1	0.197389	0.149667	1.73	1.337

	UQCRB	8	0	0.273693	0.075251	2.11	0.656
COMPLEX IV	COX5A	8	0	0.438199	0.136796	3.68	2.266
	COX4I1	8	0	0.163511	0.060022	1.23	0.380
	COX6C	8	0	0.411136	0.3722	3.36	3.019
	NDUFA4	8	0	0.291525	0.247055	2.57	2.533
	COX5B	8	0	0.218319	0.091718	1.62	0.592
	COX7A2	7	1	0.0623	0.02908	0.43	0.195
	COX6B1	8	0	1.054649	0.353936	7.98	2.432
	COX7C	8	0	0.215246	0.167467	1.97	1.930
	MT-CO2	8	0	0.058279	0.023188	0.45	0.204
COMPLEX V	ATP5F1D	8	0	0.122308	0.042978	1.05	0.792
	ATP5F1C	8	0	0.141004	0.047895	1.14	0.595
	ATP5F1B	8	0	0.787458	0.175527	6.37	3.490
	ATP5F1A	8	0	1.016675	0.402632	8.93	7.094
	ATP5IF1	7	1	0.072899	0.030805	0.57	0.337
	ATP5PB	8	0	0.062539	0.031647	0.55	0.417
FATTY ACID OXIDATION	ACAA2	8	0	0.467281	0.287802	3.88	2.829
	HADHB	8	0	0.157779	0.046768	1.19	0.338
	HADHA	8	0	0.154538	0.054823	1.13	0.251
	HADH	8	0	0.166749	0.096829	1.31	0.865
	ECHS1	8	0	0.0747	0.034038	0.56	0.292
	ECH1	8	0	0.069023	0.031908	0.57	0.338
	ETFDH	8	0	0.056609	0.015496	0.42	0.105
	ETFB	8	0	0.151318	0.075476	1.24	0.769
	ETFA	8	0	0.127043	0.035478	0.99	0.351
	ACADVL	7	1	0.01572	0.010848	0.12	0.083
	ACADSB	8	0	0.027591	0.026125	0.18	0.139
	ACADL	8	0	0.125851	0.034951	0.94	0.201
	ACADM	8	0	0.03764	0.009279	0.28	0.057
	ACADS	8	0	0.064871	0.021712	0.48	0.120
	CACT	3	5	0.00877	0.00073	0.06	0.006
	CPT2	8	0	0.009139	0.003277	0.08	0.043
	CPT1	2	6	0.00503	0.002687	0.04	0.019
PEROXISOMES	HSD17B4	8	0	0.047441	0.007484	0.36	0.089
	ACAA1B	5	3	0.09535	0.019385	0.80	0.194
	ACAA1A	6	2	0.095727	0.030506	0.64	0.151
	EHHADH	6	2	0.006485	0.00286	0.06	0.026
	ACOX3	3	5	0.00367	0.000366	0.02	0.000
	ACOX1	8	0	0.215068	0.106729	1.72	0.992
	CAT	8	0	0.311046	0.065265	2.51	1.195
PYR	PC	8	0	0.047556	0.023012	0.38	0.210
	PDHB	8	0	0.048963	0.026898	0.35	0.156
	PDHA1	8	0	0.043828	0.029479	0.31	0.176
GLUTAMINE	SLC25A12	8	0	0.044289	0.020261	0.36	0.210
	SLC25A11	4	4	0.01336	0.005944	0.11	0.053
	GLUD1	8	0	0.53751	0.188506	4.53	2.997
	GOT2	8	0	0.242289	0.10172	2.09	1.573
	GOT1	3	5	0.015193	0.010746	0.18	0.187
	GLS	8	0	0.01911	0.014291	0.13	0.079
MAS	GDP2	8	0	0.028374	0.010305	0.21	0.047
	OGC	4	4	0.01336	0.005944	0.11	0.053
	AGC2	4	4	0.01336	0.005944	0.11	0.053
	AGC1	8	0	0.044289	0.020261	0.36	0.210
	GOT2	8	0	0.242289	0.10172	2.09	1.573
	GOT1	4	4	0.011395	0.011606	0.18	0.187
	MDH2	8	0	0.627925	0.137635	5.07	2.235
	MDH1	8	0	0.018423	0.009144	0.14	0.055

Table S2. Energy metabolism enzymes in HAECs; proteomic analysis

The relative abundance (emPAI%) of proteins identified in HAECs related to the energy metabolism pathways, presented also in reference (ratio) to GAPDH abundance. Data are shown as means ± SDs from eight replicates.

HAECs				emPAI%		ratio (GAPDH)	
Process	Protein	N Analysis	N Missing	Mean	SD	Mean	SD
GLYCOLYSIS	LDHB	8	0	0.15591	0.07397	0.31	0.137
	LDHA	8	0	0.21782	0.07511	0.44	0.127
	PKM	8	0	0.42104	0.05914	0.86	0.156
	ENO1	8	0	0.50864	0.1662	1.04	0.334
	PGAM1	8	0	0.15511	0.04282	0.31	0.072
	PGK1	8	0	0.25262	0.08933	0.51	0.159
	GAPDH	8	0	0.49252	0.04737	1.00	0
	TPI1	8	0	0.20965	0.02654	0.43	0.070
	ALDOC	8	0	0.03656	0.0118	0.08	0.029
	ALDOA	8	0	0.48887	0.06515	1.00	0.148
	PFKP	8	0	0.09563	0.01302	0.20	0.037
	PFKL	8	0	0.02451	0.0093	0.05	0.015
	PFKM	8	0	0.01928	0.00526	0.04	0.012
	GPI	8	0	0.07742	0.0131	0.16	0.032
	HK1	8	0	0.04495	0.01303	0.09	0.027
TCA	FH	8	0	0.021186	0.010149	0.04	0.021
	SDHB	8	0	0.01268	0.007584	0.03	0.017
	SDHA	8	0	0.016011	0.008517	0.03	0.018
	SUCLA2	5	3	0.00769	0.003164	0.02	0.007
	SUCLG2	8	0	0.01659	0.007984	0.03	0.018
	SUCLG1	8	0	0.00824	0.005263	0.02	0.012
	OGDH	8	0	0.016171	0.007982	0.03	0.018
	IDH3G	6	2	0.019545	0.009322	0.04	0.020
	IDH3B	6	2	0.021855	0.010201	0.05	0.022
	IDH3A	7	1	0.024091	0.016914	0.05	0.039
	ACO2	8	0	0.040369	0.028848	0.08	0.061
	CS	8	0	0.059211	0.03288	0.12	0.070
COMPLEX I	NDUFV2	8	0	0.010224	0.00631	0.02	0.014
	NDUFV1	8	0	0.024789	0.018052	0.05	0.040
	NDUFS8	8	0	0.015534	0.011222	0.03	0.025
	NDUFS6	8	0	0.052031	0.030571	0.11	0.071
	NDUFS5	8	0	0.021463	0.007928	0.04	0.017
	NDUFS4	5	3	0.027844	0.012864	0.06	0.031
	NDUFS3	8	0	0.071586	0.06042	0.15	0.124
	NDUFS2	5	3	0.009606	0.005167	0.02	0.010
	NDUFS1	7	1	0.031246	0.022928	0.07	0.047
	NDUFC2	5	3	0.01637	0.007979	0.03	0.014
	NDUFB10	7	1	0.035943	0.024428	0.08	0.049
	NDUFB9	5	3	0.02468	0.009425	0.05	0.021
	NDUFB4	3	5	0.015187	0.010162	0.03	0.025
	NDUFB3	3	5	0.023997	0.013178	0.04	0.032
	NDUFA9	5	3	0.01653	0.007388	0.03	0.015
	NDUFA8	8	0	0.066446	0.048223	0.14	0.110
	NDUFA7	6	2	0.062152	0.035123	0.13	0.075
	NDUFA6	7	3	0.013156	0.006418	0.02	0.017
	NDUFA5	8	0	0.056691	0.040166	0.12	0.093
	NDUFA2	8	0	0.027329	0.013596	0.06	0.028
	NDUFA13	8	0	0.018485	0.011623	0.04	0.024
	NDUFA12	2	8	0.017	0.009235	0.03	0.019
	NDUFA10	7	3	0.025079	0.014835	0.05	0.032
COMPLEX III	CYC1	7	1	0.008869	0.005256	0.02	0.011
	UQCRC1	8	0	0.049953	0.024225	0.10	0.053
	UQCRC2	8	0	0.041833	0.024424	0.09	0.051
	UQCRH	8	0	0.063565	0.022182	0.13	0.051
	UQCRFS1	8	0	0.006954	0.001772	0.01	0.003
	UQCR10	5	3	0.038606	0.017931	0.08	0.037
	UQCRQ	6	2	0.071088	0.054398	0.15	0.124

	UQCRB	8	0	0.089091	0.051264	0.19	0.110
COMPLEX IV	COX5A	8	0	0.081259	0.016584	0.17	0.041
	COX4I1	8	0	0.022538	0.013513	0.05	0.031
	COX6C	7	1	0.029181	0.01593	0.06	0.036
	NDUFA4	8	0	0.037571	0.017381	0.08	0.037
	COX5B	8	0	0.098948	0.054913	0.20	0.114
	COX7A2	7	1	0.046941	0.006531	0.10	0.020
	COX6B1	8	0	0.273906	0.158517	0.58	0.370
	COX7C	5	3	0.046636	0.025658	0.10	0.055
	MT-CO2	8	0	0.026714	0.015991	0.06	0.035
COMPLEX V	ATP5F1D	8	0	0.016735	0.00651	0.03	0.013
	ATP5F1C	8	0	0.035705	0.022015	0.07	0.049
	ATP5F1B	8	0	0.128681	0.035037	0.27	0.082
	ATP5F1A	8	0	0.155289	0.053907	0.32	0.130
	ATP5IF1	7	1	0.027989	0.017724	0.06	0.035
	ATP5PB	7	1	0.037259	0.025898	0.08	0.052
FATTY ACID OXIDATION	ACAA2	2	6	0.00858	0.002857	0.02	0.006
	HADHB	8	0	0.074708	0.035637	0.15	0.077
	HADHA	8	0	0.059408	0.01879	0.12	0.041
	HADH	6	2	0.006247	0.002568	0.01	0.006
	ECHS1	8	0	0.047898	0.023922	0.10	0.052
	ECH1	8	0	0.017155	0.011671	0.04	0.026
	ETFDH	4	4	0.003585	0.001549	0.01	0.003
	ETFB	8	0	0.029044	0.014906	0.06	0.032
	ETFA	8	0	0.037145	0.018428	0.08	0.043
	ACADVL	7	1	0.02198	0.009559	0.05	0.021
	ACADSB	1	7	0.00346	--	0.01	--
	ACADL	0	8	--	--	--	--
	ACADM	0	8	--	--	--	--
	ACADS	3	5	0.003773		0.01	0.000
	CACT	0	8	--	--	--	--
	CPT2	2	6	0.00224	0	0.00	0.000
	CPT1	8	0	0.00993	0.004165	0.02	0.009
PEROXISOMES	HSD17B4	8	0	0.031341	0.00791	0.06	0.012
	ACAA1B	8	0	0.01312	0.008344	0.03	0.016
	ACAA1A	8	0	0.01312	0.008344	0.03	0.016
	EHHADH	0	8	--	--	--	--
	ACOX3	5	3	0.00471	0.002164	0.01	0.004
	ACOX1	7	1	0.013649	0.009325	0.03	0.021
	CAT	8	0	0.013816	0.008	0.03	0.018
PYR	PC	0	8	--	--	--	--
	PDHB	8	0	0.036033	0.014424	0.08	0.036
	PDHA1	7	1	0.031743	0.025203	0.06	0.059
GLUTAMINE	SLC25A12	1	7	0.00672	--	0.01	--
	SLC25A11	6	2	0.005557	0.002598	0.01	0.006
	GLUD1	8	0	0.054739	0.015756	0.11	0.034
	GOT2	8	0	0.043325	0.025849	0.09	0.055
	GOT1	8	0	0.028589	0.016313	0.06	0.031
	GLS	7	1	0.021433	0.013041	0.05	0.028
MAS	GDP2	8	0	0.00486	0.002824	0.01	0.006
	OGC	6	2	0.005557	0.002598	0.01	0.006
	AGC2	6	2	0.005557	0.002598	0.01	0.006
	AGC1	1	7	0.00672	--	0.01	--
	GOT2	8	0	0.043325	0.025849	0.09	0.055
	GOT1	8	0	0.028589	0.016313	0.06	0.031
	MDH2	8	0	0.197218	0.063146	0.41	0.155
	MDH1	8	0	0.034589	0.01206	0.07	0.022

Table S3. Acylcarnitines levels in LSECs

Acylcarnitine levels in LSECs incubated for 2 h in KHB supplemented with glucose (2.5 mM) and L-carnitine (50 μM). Data are shown in pmol/μg of protein as means ± SDs from four independent experiments.

	**CTR**	**BSA**	**BSA_ETO**	**PA**	**PA_ETO**
**Carnitine**	67.1 ± 38.22	88.7 ± 39.03	129.3 ± 38.67	116.1 ± 74.32	105.5 ± 59.72
**Acetylcarnitine**	180.9 ± 52.53	178.3 ± 41.64	144.3 ± 41.02	224.2 ± 78.39	221.9 ± 39.61
**Propionylcarnitine**	79.2 ± 17.64	94.9 ± 26.08	101.8 ± 31.04	51.0 ± 24.74	51.4 ± 12.69
**Butyrylcarnitine**	3.5 ± 1.94	3.4 ± 2.25	2.6 ± 2.11	4.7 ± 2.42	14.6 ± 16.80
**Valerylcarnitine**	2.9 ± 1.67	4.4 ± 3.25	4.3 ± 3.44	1.6 ± 0.91	1.8 ± 0.55
**Hexanoylcarnitine**	2.0 ± 0.94	2.2 ± 1.55	1.7 ± 1.44	2.2 ± 1.03	2.9 ± 0.48
**Octanoylcarnitine**	0.4 ± 0.24	0.5 ± 0.36	0.4 ± 0.35	0.6 ± 0.31	0.6 ± 0.15
**Decanoylcarnitine**	0.4 ± 0.26	0.4 ± 0.35	0.3 ± 0.35	0.9 ± 0.47	0.8 ± 0.25
**Lauroylcarnitine**	0.7 ± 0.39	0.6 ± 0.36	0.3 ± 0.25	2.1 ± 0.99	2.1 ± 0.49
**Myristoylcarnitine**	3.6 ± 1.65	2.7 ± 1.13	1.4 ± 0.62	12.2 ± 6.25	13.1 ± 2.68
**Palmitoylcarnitine**	658.6 ± 115.00	438.6 ± 76.78	190.9 ± 39.18	1402.6 ± 716.56	1614.2 ± 79.81
**Stearoylcarnitine**	15.3 ± 1.28	7.6 ± 2.55	3.6 ± 0.98	4.6 ± 2.58	4.5 ± 0.54
**Oleoylcarnitine**	19.0 ± 4.30	10.8 ± 2.21	5.6 ± 1.60	1.7 ± 0.87	1.7 ± 0.28
**Linoleoylcarnitine**	110.6 ± 35.58	79.3 ± 20.21	40.7 ± 11.59	11.5 ± 6.69	11.2 ± 1.90
**Total**	1144.3 ± 258.04	912.5 ± 124.91	627.2 ± 73.52	1836.0 ± 882.88	2046.2 ± 188.99
