# Supplementary material for: Liver sinusoidal endothelial cells rely on oxidative phosphorylation but avoid processing long-chain fatty acids in their mitochondria
Source: Cell Mol Biol Lett. 2024 May 9;29:67. doi: 10.1186/s11658-024-00584-8 (PMC11084093; doi:10.1186/s11658-024-00584-8)
Supplement: Supplementary file 2 — Additional file 2: Key resources table. [file 11658_2024_584_MOESM2_ESM.pdf]

KEY RESOURCES TABLE

REAGENT or RESOURCE	SOURCE	IDENTIFIER
**Antibodies**		
Recombinant Anti-CPT1A antibody [EPR21843-71-2F]	abcam	Cat#ab234111;
Mouse IgG kappa binding protein conjugated to horse radish peroxidase (m-IgGκ BP-HRP)	Santa Cruz Biotechnology	Cat#sc-516102
**Chemicals, peptides, and recombinant proteins**		
Acetonitrile	J. T. Baker	Cat# 9821.1000; CAS: 75-05-8
Acetyl-DL-carnitine hydrochloride	Sigma-Aldrich	Cat# A1509; CAS: 2504-11-2
Ammonium bicarbonate	Sigma-Aldrich	Cat# 09830; CAS: 1066-33-7
Ammonium molybdate	Sigma-Aldrich	Cat# 09878; CAS: 12054-85-2
Antimycin A	Sigma-Aldrich	Cat#A8674; CAS: 1397-94-0
Bovine Serum Albumin (BSA) Fraction V, heat shock, fatty acid-free	Roche	Cat# 03117057001; CAS: 9048-46-8
BPTES (glutaminase 1 inhibitor (bis-2-[5-phenylacetamido-1,3,4-thiadiazol-2-yl]ethyl sulphide)	Tocris Bioscience	Cat# 5301; CAS: 314045-39-1
Butyric acid	Sigma-Aldrich	Cat# B103500; CAS: 107-92-6
Butyryl-L-carnitine	Sigma-Aldrich	Cat# 08984; CAS: 25576-40-3
L-carnitine hydrochloride	Sigma-Aldrich	Cat# C0283; CAS: 6645-46-1
CB-839	Cayman Chemical	Cat#22038; CAS: 1439399-58-2
CD146 MicroBeads	Miltenyi Biotec	Cat# 130-092-007
Decanoyl-L-carnitine	Sigma-Aldrich	Cat# 50637; CAS: 3992-45-8
2-Deoxy-D-glucose (2DG)	Sigma-Aldrich	Cat# D8375, CAS: 154-17-6
Dithiothreitol	BioShop	Cat# DTT001.5; CAS: 3483-12-3
Etomoxir sodium salt hydrate	Sigma-Aldrich	Cat# E1905, CAS: 828934-41-4
FCCP (Carbonyl cyanide 4-[trifluoromethoxy]phenylhydrazone)	Sigma-Aldrich	Cat#C2920; CAS: 370-86-5
Formic acid, 0.1% in water	ThermoFisher Scientific	Cat# 85178
Glucose	MERCK	Cat# 1.08337.1000; CAS: 50-99-7
Glutamine	Sigma-Aldrich	Cat#G3126; CAS: 56-85-9
Glutaraldehyde solution	Sigma-Aldrich	Cat#340855; CAS: 111-30-8
HEPES 4-(2-hydroxyethyl)-1-piperazineethanesulfonic acid	Sigma-Aldrich	Cat#H4034; CAS: 7365-45-9
Hexanoyl-L-carnitine	Sigma-Aldrich	Cat# 07439; CAS: 22671-29-0
Hoechst 33342	Invitrogen	Cat# H3570; CAS: 23491-52-3
Hydrogen peroxide	EMD Millipore Corp.	Cat# 386790; CAS: 7722-84-1
Insulin solution human	Sigma-Aldrich	Cat# I9278; CAS: 11061-68-0
Iodoacetamide	Sigma-Aldrich	Cat# I6125; CAS: 144-48-9
Iodoacetic acid	Sigma-Aldrich	Cat# I4386; CAS: 64-69-7
Lauroyl-L-carnitine	Sigma-Aldrich	Cat# 39953; CAS: 25518-54-1
Liberase TM Research Grade	Roche	Cat# 14040091
Linoleoyl-L-carnitine (chloride)	Cayman Chemical	Cat# 26560; CAS: 173686-75-4
MitoTracker Green FM	Invitrogen	Cat# 130-092-007
Myristoyl-L-carnitine	Sigma-Aldrich	Cat# 61367; CAS: 25597-07-3
Myristoyl-L-carnitine-(N,N,N-trimethyl-d9)	Sigma-Aldrich	Cat# 94346; CAS: 2245713-18-0
MitoTracker Green FM	Invitrogen	Cat# 14040091
Octanoic acid	Sigma-Aldrich	Cat# O3907; CAS: 124-07-2
Octanoyl-L-carnitine	Sigma-Aldrich	Cat# 50892; CAS: 25243-95-2
Oleoyl-L-carnitine	Sigma-Aldrich	Cat# 19945; CAS: 38677-66-6

Oligomycin	EMD Millipore Corp.	Cat#495455; CAS: 1404-19-9
Oxfenicine (4-Hydroxy-L-phenylglycine)	Sigma-Aldrich	Cat# 56160, CAS: 32462-30-9
Palmitic acid-d31	Sigma-Aldrich	Cat# 366897, CAS: 39756-30-4
Palmitoyl-L-carnitine	Sigma-Aldrich	Cat# P1645; CAS: 18877-64-0
Percoll	Sigma-Aldrich	Cat# GE17-0891-01
Perhexiline maleate salt	Sigma-Aldrich	Cat# SML0120, CAS: 6724-53-4
Pierce Formic Acid, LC-MS Grade	ThermoFisher Scientific	Cat# 85178, CAS: 64-18-6
Pierce 0.1% Formic Acid (v/v) in Acetonitrile, LC-MS Grade	ThermoFisher Scientific	Cat# 85175
Propionyl-L-carnitine	Sigma-Aldrich	Cat# 42602; CAS: 20064-19-1
Protease and Phosphatase Inhibitor Cocktail (PiC)	Sigma-Aldrich	Cat# MSSAFE
Rotenone	Sigma-Aldrich	Cat#R8875; CAS: 83-79-4
Sodium pyruvate	Sigma-Aldrich	Cat#P5280; CAS: 113-24-6
Stearoyl-L-carnitine	Sigma-Aldrich	Cat# 61229; CAS: 25597-09-5
Thiourea	BioShop	Cat# THI777.100; CAS: 62-56-6
Trifluoroacetic acid	Sigma-Aldrich	Cat# 302031; CAS: 76-05-1
Tris-HCl	BioShop	Cat# TRS002.500; CAS: 1185-53-1
Trypsin, sequencing grade	Promega	Cat# V5111
UK-5099	Tocris Bioscience	Cat#4186; CAS: 56396-35-1
Urea	BioShop	Cat# URE001.500; CAS: 57-13-6
Valeryl-L-carnitine	Sigma-Aldrich	Cat# 04265; CAS: 40225-14-7
**Critical commercial assays**		
Agilent Technologies XF Palmitate BSA FAO Substrate	Agilent Technologies	Cat# 102720-100
ATPlite 1-Step Luminescence Assay System	PerkinElmer	Cat# 6016736
Bicinchoninic Acid Kit for Protein Determination (BCA)	Sigma-Aldrich	Cat# BCA1
Bradford Assay for Protein Determination	Bio-Rad	Cat# 5000205
Free Fatty Acid Quantification Kit	Sigma-Aldrich	Cat# MAK044
Pierce BCA Protein Assay Kit	Thermo Fisher Scientific	Cat# 23227
TGX Stain-Free FastCast Acrylamide Kit, 7.5%	Bio-Rad	Cat# 1610181
**Experimental models: Cell lines**		
Human aortic endothelial cells (HAEC)	Lonza	CC-2535
Human breast cancer adenocarcinoma MCF-7 cells	American Type Culture Collection	HTB-22
Human breast cancer adenocarcinoma MCF-7 cells	American Type Culture Collection	HTB-26
Human lung microvascular endothelial cells	Cell Applications	540-05a
**Experimental models: organisms/strains**		
C57BL/6 mice (male)	Medical University of Bialystok, Poland	
**Software and algorithms**		
OriginPro 2022b	OriginLab Corporation, Northampton, MA, USA	https://www.originlab.com/
Shiny GO 0.77	South Dakota State University (SDSU)	http://bioinformatics.sdstate.edu/go/
BioRender	www.biorender.com	

Other		
Agilent Seahorse XF Base Medium Minimal DMEM	Agilent	Cat# 102353-100
DPBS (Dulbecco's modified Eagle's medium) without Calcium and Magnesium	Thermo Scientific	Cat# 14190250
DPBS (Dulbecco's modified Eagle's medium) with Calcium and Magnesium	Thermo Scientific	Cat# 14040091
Eagle medium	Hirszfeld Institute of Immunology and Experimental Therapy, Polish Academy of Sciences	Cat# 20.59.52.0
EGM-2 BulletKit Medium	Lonza, Basel, Switzerland	Cat# CC-3162
Fetal bovine serum (FBS)	Thermo Fisher Scientific	Cat# A3160402
MECGM (Microvascular Endothelial Cell Growth Medium)	Cell Applications	Cat# 111-500
MEM (Minimal Essential Medium) Non-essential Amino Acid Solution	Sigma-Aldrich	Cat# M7145
RPMI 1640 medium	Thermo Scientific	Cat# 72400047

KEY RESOURCES TABLE

Lead contact

Further information and requests for resources and reagents should be directed to and will be fulfilled by the lead contact, Patrycja Kaczara (patrycja.kaczara@jcet.eu)

Material availability

This study did not generate new unique reagents

Data and code availability

All data reported in this paper will be shared by the lead contact upon request

This paper does not report original code

Any additional information required to reanalyze the data in this paper is available from the lead contact upon request.
